# Mining Time-Resolved Functional Brain Graphs to an EEG-Based Chronnectomic Brain Aged Index (CBAI)

**DOI:** 10.3389/fnhum.2017.00423

**Published:** 2017-09-07

**Authors:** Stavros I. Dimitriadis, Christos I. Salis

**Affiliations:** ^1^Institute of Psychological Medicine and Clinical Neurosciences, Cardiff University School of Medicine Cardiff, United Kingdom; ^2^Cardiff University Brain Research Imaging Center (CUBRIC), School of Psychology, Cardiff University Cardiff, United Kingdom; ^3^Neuroinformatics Group, Cardiff University Brain Research Imaging Center (CUBRIC), School of Psychology, Cardiff University Cardiff, United Kingdom; ^4^Department of Informatics and Telecommunications Engineering, University of Western Macedonia Kozani, Greece

**Keywords:** EEG, time-varying network analysis, chronnectomics, dominant coupling modes, cross-frequency coupling, maturation index, signal processing, symbolic analysis

## Abstract

The brain at rest consists of spatially and temporal distributed but functionally connected regions that called intrinsic connectivity networks (ICNs). Resting state electroencephalography (rs-EEG) is a way to characterize brain networks without confounds associated with task EEG such as task difficulty and performance. A novel framework of how to study dynamic functional connectivity under the notion of functional connectivity microstates (FCμstates) and symbolic dynamics is further discussed. Furthermore, we introduced a way to construct a single integrated dynamic functional connectivity graph (IDFCG) that preserves both the strength of the connections between every pair of sensors but also the type of dominant intrinsic coupling modes (DICM). The whole methodology is demonstrated in a significant and unexplored task for EEG which is the definition of an objective Chronnectomic Brain Aged index (CBAI) extracted from resting-state data (*N* = 94 subjects) with both eyes-open and eyes-closed conditions. Novel features have been defined based on symbolic dynamics and the notion of DICM and FCμstates. The transition rate of FCμstates, the symbolic dynamics based on the evolution of FCμstates (the Markovian Entropy, the complexity index), the probability distribution of DICM, the novel Flexibility Index that captures the dynamic reconfiguration of DICM per pair of EEG sensors and the relative signal power constitute a valuable pool of features that can build the proposed CBAI. Here we applied a feature selection technique and Extreme Learning Machine (ELM) classifier to discriminate young adults from middle-aged and a Support Vector Regressor to build a linear model of the actual age based on EEG-based spatio-temporal features. The most significant type of features for both prediction of age and discrimination of young vs. adults age groups was the dynamic reconfiguration of dominant coupling modes derived from a subset of EEG sensor pairs. Specifically, our results revealed a very high prediction of age for eyes-open (*R*^2^ = 0.60; y = 0.79x + 8.03) and lower for eyes-closed (*R*^2^ = 0.48; y = 0.71x + 10.91) while we succeeded to correctly classify young vs. middle-age group with 97.8% accuracy in eyes-open and 87.2% for eyes-closed. Our results were reproduced also in a second dataset for further external validation of the whole analysis. The proposed methodology proved valuable for the characterization of the intrinsic properties of dynamic functional connectivity through the age untangling developmental differences using EEG resting-state recordings.

## Introduction

Functional networks can be defined as spatio-temporal correlation of brain areas which normally involved in a task and are also observed when subjects are not performing a specific task called resting-state (Biswal et al., 1995).

Many neuroimaging efforts in functional magnetic resonance imaging (fMRI) have focused from studying cognitive subsystems directly linked to the experimental paradigms focusing on one or more subsystems (Turk-Browne, 2013). Targeted cognitive domains are vision, language, memory, and emotion. Recent studies focused to assess individual differences in functional connectivity across multiple whole-brain networks (Thomason et al., 2008). Subsequently, an increasing number of studies using rs-fMRI data are showing reproducibility and reliability of features and connectivity patterns extracted from the whole brain (Damoiseaux et al., 2006; Thomason et al., 2008; Shehzad et al., 2009; Van Dijk et al., 2010; Zuo et al., 2010; Song et al., 2012).

It is high popular in fMRI studies to use machine learning techniques in corporation with fMRI BOLD activity and with brain networks. Especially, support Vector Machines (SVMs) have become widely used for different approaches due to their advantage to manipulate high-dimensional data to the direction of classification and predictive accuracy (Schölkopf and Smola, 2001; Ben-Hur and Weston, 2010; Meier et al., 2012). The backbone of fMRI-based studies adopted a seed-based connectivity analysis, independent component analysis (ICA), graph and network theory methods, and machine learning techniques that include feature extraction algorithms and various classifiers like SVM. A variety of neuroimaging studies have clearly shown that machine learning algorithms applying to human recordings can extract novel insights human brain activity (Haynes and Rees, 2005; Cohen et al., 2011).

Resting state fMRI neuroimaging data has been shown to perform well in two significant tasks: classification and prediction. Craddock et al. (2009) adopted resting state functional connectivity fMRI (rs-fc-fMRI) data to successfully distinguish individuals with major depressive disorder from healthy controls with a high 95% accuracy using a linear classifier and appropriate feature selection algorithm. Supekar et al. (2009) achieved a classification of individuals as either children or young-adults with 90% accuracy, using SVM classifier. Also Shen et al. (2010) performed accuracy equal to 81% in the discrimination between schizophrenic patients and healthy controls, using SVM classifier and a 92% accuracy was achieved using a *C*-means clustering classifier with locally linear embedding (LLE) feature selection algorithm.

Last few years, a series of (rs-fc)—fMRI papers appeared in the literature focusing on defining an objective maturity index based on dynamic networks, discrimination of age- groups working thought out the development or the whole range of human age across the lifespan (8–88 age). Dosenbach et al. (2010) used static networks from a large population and SVM classifier, achieving 91% accuracy for classification of individuals as either children or adults, and also they predicted functional maturity for each participant's brain resting-state functional brain network using a support vector machine regression (SVR). Vergun et al. (2013) studied the prediction of age (10–70 age) and discrimination of young vs. old with static functional connectivity graphs (FCG) based on rs-fc-fMRI. Following, a feature selection and a linear SVR, they succeeded an acceptable prediction of age (*R*^2^ = 0.419) while they performed 84% classification for the discrimination of two age-groups using SVM. A recent study demonstrated a high correlation of age with a more dynamic perspective of inter-network interactions on resting-state with fMRI (Qin et al., 2015). Furthermore, Betzel et al. (2014) modeled dynamic functional connectivity at resting-state with fMRI through the lifespan. They revealed functional changes in specific systems like in control, default mode, saliency/ventral attention, dorsal attention, and visual networks which become less cohesive through the age, while both the density and the strength of the structural connections decrease through the age.

Another study complements the previous rs-fc-fMRI explorations revealing a modification of dynamic couplings to both constrain and maximize functional variability across the lifespan in coordination to cognitive and behavioral demands (Hutchison and Morton, 2015).

A lot of research explored developmental differences across the lifespan and between specific age groups at resting-state (spontaneous activity) with fMRI adopting both static and dynamic connectivity analysis, as afore mentioned. In contrast, only a few studies attempted to analyze spontaneous (resting-state) electrocortical activity across the lifespan via the notion of dynamic network analysis. In Kurth et al. (2015), they explored the interaction of sleep and maturation across early childhood. A recent study examined electrocortical maturation at children from early (7 years) to late childhood (11 years) where they found topological differences especially in α frequency band (Miskovic et al., 2015).

Brain signal recorded via electroencephalography (EEG) is a complex signal containing the activity with different frequency profile. Apart from power spectrum analysis of the brain signals using Fourier or wavelet transform to reveal the amplitude modulations, it is important one to explore the different type of interactions. Intrinsic coupling modes (ICMs) in spontaneous activity reflect two basic complementary coupling mechanisms, the phase coupling of the bandpass filtered brain signals and the coupling of the signal envelopes (Engel et al., 2013). Apart from exploring the ICMs by taking into account the functional coupling of signals with the same frequency, it is more than important to explore their cross-frequency interactions (Jirsa and Müller, 2013; Dimitriadis et al., 2015b,c). Going one step further, the multiplexity of the human brain functionality can be detected via brain connectivity only if all the interactions are studying together. For that reason, it is significant to define the dominant type of interaction between every pair of brain areas at every small time-window across the experimental time. We hypothesize that the dynamic reconfiguration of dominant coupling modes is a key mechanism that can be linked with the flexibility of the human brain at first and secondly to its actual age (Buzsáki and Draguhn, 2004; Buzsaki, 2010; Buzsaki et al., 2013).

In this paper, we go one step further by modeling dynamic functional connectivity patterns (Dimitriadis et al., 2013a,b, 2015a,b) at resting-state using a dataset of 94 subjects from 18 to 60 years. The final outcome of this analysis is a symbolic time series that encodes the metastability of individual brain state. We first design a codebook of prototypical network microstates and then we assign each of this instantaneous connectivity pattern to the most similar code symbol (e.g., functional connectivity graph—FCG) (Dimitriadis et al., 2013a, 2015a). In this way, a unique symbolic time series derived from each individual where each symbol corresponds to one of the predefined prototypical connectivity patterns. The evolution of these symbols-patterns encapsulates significant state transitions.

Our analysis is simultaneously unique because attempts to construct a dynamic functional connectivity that integrates both within frequency interactions and also between frequencies called cross-frequency interactions (Jirsa and Müller, 2013). We followed an appropriate surrogate analysis and we assigned to each pair of sensors across each quasi-instantaneous FCG at any point t, the dominant type of interactions (Engel et al., 2013; Dimitriadis et al., 2016a). With this approach apart from the weight that describes the strength of functional coupling between two temporal segments from two spatially apart EEG sensors, we keep the type of preferred interaction. The dominant intrinsic coupling mode (DICM) could be within α frequency band or between δ-γ as a cross-frequency coupling. The final outcome of this approach is an integrated dynamic functional connectivity graph (IDFCG) that incorporates both the weight and the type of DICM. The whole approach is unique and first presented here.

The proposed methodological scheme has two distinct ways of analyzing the dynamic functional connectivity patterns based on IDFCG. The first approach constitutes novel contributions to an emerging neuroimaging field called chronnectomics (Calhoun et al., 2014). The concept of chronnectome is the incorporation of a dynamic view of functional brain connectivity networks and the evolution of revisiting distinct spatio-temporal brain states (functional connectivity microstates—FCμstates). The second approach is a novel way to understand how brain areas change preferred type of interactions over time even at resting-state, in the absence of external stimuli. The dynamic reconfiguration of dominant intrinsic coupling modes can be a valuable tool to describe how flexible is a brain, probably a unique characteristic for each individual. To the best of our knowledge, this is the first time that such an approach is exploited for the purposes of defining a chronnectomic brain maturation index (CBMI) that can predict the actual age of an individual.

## Materials and methods

In this section, we describe the two EEG datasets used here to validate the proposed developmental index. The main EEG dataset is free and available while the second is part of a previous study and was used as an external validation scheme. Additionally, we described on this section, how integrated dynamic functional connectivity networks (IDFCG) was constructed and how it was modeled via the proposed scheme. Complementary, we give a seminar description of different features that can be extracted from the IDFCG called chronnectomics.

### EEG recordings

Scalp EEG signals were gathered from the free online database PhysioNet BCI (Database physionet BCI^1^) (Schalk et al., 2004). The database consists of *N* = 94 healthy subjects recorded in two different baseline conditions, i.e., 1-min eyes-open (EO) resting state and 1-min eyes-closed (EC) resting state. In each condition, subjects were comfortably seated on a reclining chair in a dimly lit room. During EO they were asked to avoid ocular blinks in order to reduce signal contamination. The EEG data were recorded with a 64-channel system (BCI2000 system (BCI2000 system) with an original sampling rate of 160 Hz. From the 94 healthy subjects 39 were male with mean_age = 37.69 και std_age = 12.63 and the rest 55 were female with mean_age = 39.4 και std_age = 10.22. The range of age in the training sample was between 19 and 67.

We used a second EEG dataset for external validation of the proposed scheme based on 100 subjects from ages 18—60 from the Human Brain Institute (HBI) Normative Database using 19 EEG channels and the first min from the three recording min of eyes-open and eyes-closed condition.

### Preprocessing

**Figure 1:**
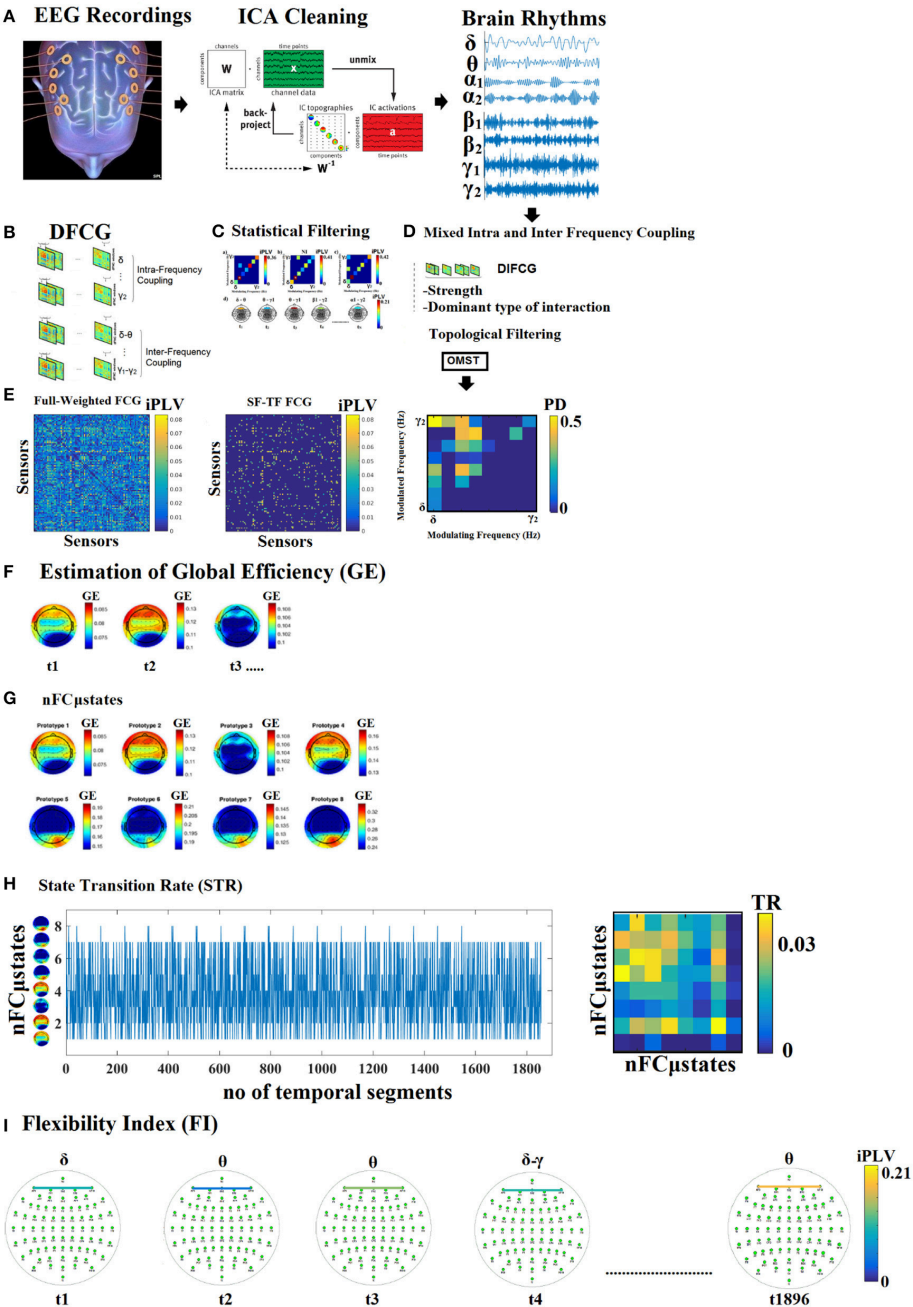
Outline of our methodology. From multiple versions of dynamic functional connectivity graphs to a single integrated DIFCG. **(A)** ICA cleaning of EEG recordings, projected from ICA space to EEG sensor space and filtered in the predefined frequency bands. **(B)** From multichannel recordings to multiple versions of dynamic functional connectivity graphs (DFCG) linked to intra and inter-frequency couplings. **(C)** An example of how to assign a dominant type of interactions among intra and cross-frequency couplings to every possible pair of sensors. **(D)** The proposed scheme derived to a single integrated dynamic functional connectivity graph (IDFCG) which finally fed to a. **(E)** Data-driven topological filtering scheme based on orthogonal minimal spanning tree (OMST). **(F)** Estimation of nodal network metric time series based on GE over the DIFCG. **(G)** The outcome of NNMF-NG algorithm is the eight nFCGμstates for eyes-open condition. **(H)** State transition vectors for a subject (age = 18). Assigned states are plotted at every time point that corresponds to the center of the sliding window while the Transition Matrix of the state vector for the same example is given. **(I)** Demonstration of how the dominant coupling mode changes over experimental time in both strength and type of dominant coupling mode.

Ongoing activity was corrected for artifacts through a two-step procedure implemented in Matlab (The MathWorks, Inc., Natick, MA, USA) and Fieldtrip (Oostenveld et al., 2011). Line noise was first removed using a notch filter at 60 Hz and the single-subject data was whitened and reduced in dimensionality by means of Principal Component Analysis (PCA) with a threshold corresponding to 95% of total variance (Delorme and Makeig, 2004; Antonakakis et al., 2013). The resulting signals were submitted to ICA using the extended Infomax algorithm as implemented in EEGLAB (Delorme and Makeig, 2004). A given independent component was considered to reflect ocular or cardiac artifacts if more than 30% of its z-score kurtosis or skewness values, respectively, were outside ±2 of the distribution mean (Antonakakis et al., 2013; Dimitriadis et al., 2015a). The remaining ICs were used to reconstruct a relatively artifact-free signal. The average number of artefactual ICs was 5.5 for eyes-closed and 5.1 for the eyes-open condition (see Figure 1A).

### Functional connectivity

Here, functional connectivity was examined among the following eight brain rhythms of the typical sub-bands of electrophysiological neural signals {δ, θ, α_1_, α_2_, β_1_, β_2_, γ1, γ2}, defined respectively within the ranges {0.5–4; 4–8; 8–10; 10–13; 13–20; 20–30; 30–48; 52–70 Hz}. We adopted a 3rd order Butterworth filters applied in a zero-phase mode to get the characteristic brain rhythms (see Figure 1A).

### Dynamic iPLV estimates: the time-varying integrated iPLV graph (^TVI^iPLV graph)

The goal of the analytic procedures described in this section is to understand the repertoire of phase-to-phase interactions and their temporal evolution, while taking into account the quasi-instantaneous spatiotemporal distribution of iPLV estimates. This was achieved by computing one set of iPLV estimates within each of a series of sliding overlapping windows spanning the entire 1-min continuous EEG recording for both eyes-open and closed condition. The width of the temporal window was set equal to the duration of 10 cycles of δ brain rhythm, as an adequate number to capture the dynamics of every brain rhythm (fast and slows, Dimitriadis et al., 2013a). The center of the stepping window moved forwards every 20 ms and both intra and inter-frequency interactions within and between every possible pair of frequencies were reestimated leading to a quasi-stable in time static iPLV graph. In this manner, a series of 1,856 of iPLV graph estimates were computed per condition, frequencies (8 within frequency + 28 possible pairs of cross-frequency interactions = 36 combinations) and for each participant.

This procedure, the implementation details of which can be found elsewhere (Dimitriadis et al., 2010, 2012, 2013b, 2015a,c), resulted in eight time-varying iPLV graphs per participant (^TV^iPLV) for within frequency bands (Section 1 in Supplementary Material) and 28 time-varying iPLV graphs per participant (^TV^iPLV) for each possible cross-frequency pair (Section 2 in Supplementary Material), each serving as an instantaneous snapshot of the surface network. ^TV^iPLV tabulates iPLV estimates between every possible pair of sensors. For each subject, a 4D tensor [frequencies bands (36) × slides (1,856) × sensors (64) × sensors (64)] was created for each condition integrating subject-specific spatio-temporal phase interactions (Figure 1B).

Afterward, we applied surrogate analysis in order to reveal the dominant type of interaction for each pair of EEG sensors and at each snapshot of the ^TV^iPLV (Figure 1C—Section 3 in Supplementary Material). Finally, we tabulated both the strength and the type of dominant coupling mode in 2 3D tensor [slides (1,856) × sensors (64) × sensors (64)], one that keeps the strength of the coupling based on iPLV and a second one for keeping the dominant coupling of interaction using integers from 1 up to 36 {1 for δ, 2 for θ,…,8 for γ_2_, 9 for δ−θ,…, 36 for β_2_ − γ_2_} (Figure 1D). The notion of phase-to-amplitude cross-frequency coupling (CFC) estimator has been used also in our previous studies (Dimitriadis et al., 2015b, 2016a,b,c, 2017).

### A data-driven topological filtering scheme based on orthogonal minimal spanning trees (OMSTs)

On the previous section, we applied statistical filtering approach in order to explore: (a) if a given iPLV value differed from what would be expected by chance alone, (b) if a non-zero iPLV corresponded to non-spurious coupling, and (c) to integrate dominant intrinsic coupling modes into a single graph called dynamic integrated functional connectivity graph (DIFCG). Apart from statistical filtering approach, it is important to adopt a data-driven topological filtering approach in order to reveal the backbone of the network topology over the increment of information flow.

The adopted topological filtering scheme based on orthogonal minimal spanning trees (OMST) (Figure 1D). Usage of the orthogonal MST graph leads to a better sampling of brain network preserving the advantage of MST that connects the whole network with minimum cost without introducing cycles and without differentiated strong from weak connections (Dimitriadis et al., 2017). For further details see also section 4 in Supplementay Material.

The outcome of both statistical and topological filtering scheme is demonstrated in Figure 1E for a static functional connectivity graph (FCG) from δ frequency of the first subject in the database. The resulting functional connectivity graph is sparser compared to the full-weighted (FCG) derived via the bivariate iPLV connectivity estimator. The FCG with dimension sensors x sensors illustrates the strength of the coupling in the surviving set of connections while the comodulogram demonstrates the probability distribution (PD) of the dominant coupling modes. The main diagonal refers to the PD of the within frequency interactions while the off-diagonal to the PD of the cross-frequency coupling namely the phase-to-amplitude couplings. The horizontal x-axis refer to how each the phase of the lower brain rhythms modulates the amplitude of the higher frequencies.

### Network metric time series (NMTS)

After applying the statistical and topological filtering approach, we estimated the global efficiency (GE) for each node and across time. This approach leads to a 2D matrix of dimensions 64 (sensors) × 1,856 (temporal segments) for each subject. This matrix tabulates the fluctuations of GE for each node and can be seen as network metric time series (NMTS) that keeps the importance of each node for integration of information (Dimitriadis et al., 2013a,b).

The estimation of GE based on the notion of shortest path length. However, we want to favor the selection of strongest functional couplings and for that reason the weights original functional connectivity graphs are inverted (1./FCG). A connection with strong coupling strength means that those two areas are functionally closed even in the case that are distant anatomically. By inverting the original weights, shortest path lengths between every pair of nodes will capture the strongest connections under the constraint of minimizing the overall distance cost. Distance cost (*d*_*ij*_) of a shortest path length is defined the total sum of weights of the path that connects two nodes, here two EEG sensors.

Network GE reflects the overall efficiency of parallel information transfer within the entire set of 64 sensors and was estimated as the average sensor-specific GE value over all sensors using the following formula:

(1) $$\begin{array}{l} GE=\frac{1}{N}\sum_{i\in N}\frac{\sum_{j\in N,\;j\neq i}{\left(d_{ij}\right)}^{-1}}{N-1} \end{array}$$

where *d*_*ij*_ denotes the distance of the shortest path length between target node *i* and the rest *N* − 1 *j* nodes. Here, we estimated the nodal *GE* which leads to 64 values per time-instant functional connectivity graph.

The derived 2D matrix based on nodal NMTS of GE will be modeled with the proposed method that is described in the following section. The first time instances of the topology of nodal GE is illustrated in Figure 1F.

### A non-negative matric factorization (NNMF)—vector-quantization (VQ) modeling of dynamic functional connectivity graphs

This subsection serves as a brief introduction to our symbolization scheme, presented in greater details elsewhere (Dimitriadis et al., 2011, 2012, 2013a,b). The dynamic functional connectivity patterns can be modeled as prototypical functional connectivity microstates (FCμstates). In a recent study, we demonstrated a better modeling of dynamic functional connectivity graphs (DFCG) based on vector quantization approach (Dimitriadis et al., 2013a), if a preprocessing is added. The algorithm called non-negative matrix factorization (NNMF) (Marimpis et al., 2016). Here, we employed the NNMF as a first step for re-parameterization of the dynamic functional connectivity graph (DFCG) within a reduced space. This reduced space keeps the most informative ingredients for a parsimonious representation of the original connectivity patterns. Within this reduced NNMF-based low-dimensional space, a codebook of *k* prototypical functional connectivity states (i.e., connectivity microstates) is first designed by applying the neural-gas algorithm (Laskaris and Ioannides, 2001). This algorithm is an artificial neural network model, which converges efficiently to a small number *k* of codebook vectors, using a stochastic gradient descent procedure with a soft-max adaptation rule that minimizes the average distortion error (Martinetz et al., 1993).

In this way, the bulk of information contained in the time series of connectivity patterns is represented, in a parsimonious way, by a partition matrix *U*, with elements *u*_*ij*_ indicating the assignment of input connectivity patterns to code vectors. Following the inverse procedure, we can rebuild a given time series from the *k* code vectors, with a small reconstruction error *E*. The selection of parameter *k* reflects the trade-off between fidelity and compression level. In this work, it was set as the smaller value to achieve a reconstruction error lower than 4%. Finally, we need to mention that inside the codebook design there is an algorithmic seriation procedure that “ranks” the symbols. As a consequence, the symbolic time series closely follows the underlying functional connectivity dynamics. The neural-gas algorithm applied to the output of NNMF is called vector-quantization scheme (VQ) and the whole proposed scheme (NNMF-VQ). The derived symbolic times series that keep the information of network FCμstates (nFCμstates) are called hereafter as STS^NNMF-VQ^. The combination of NNMF-VQ gives a lower distortion error compared to the VQ alone.

**Figure 2:**
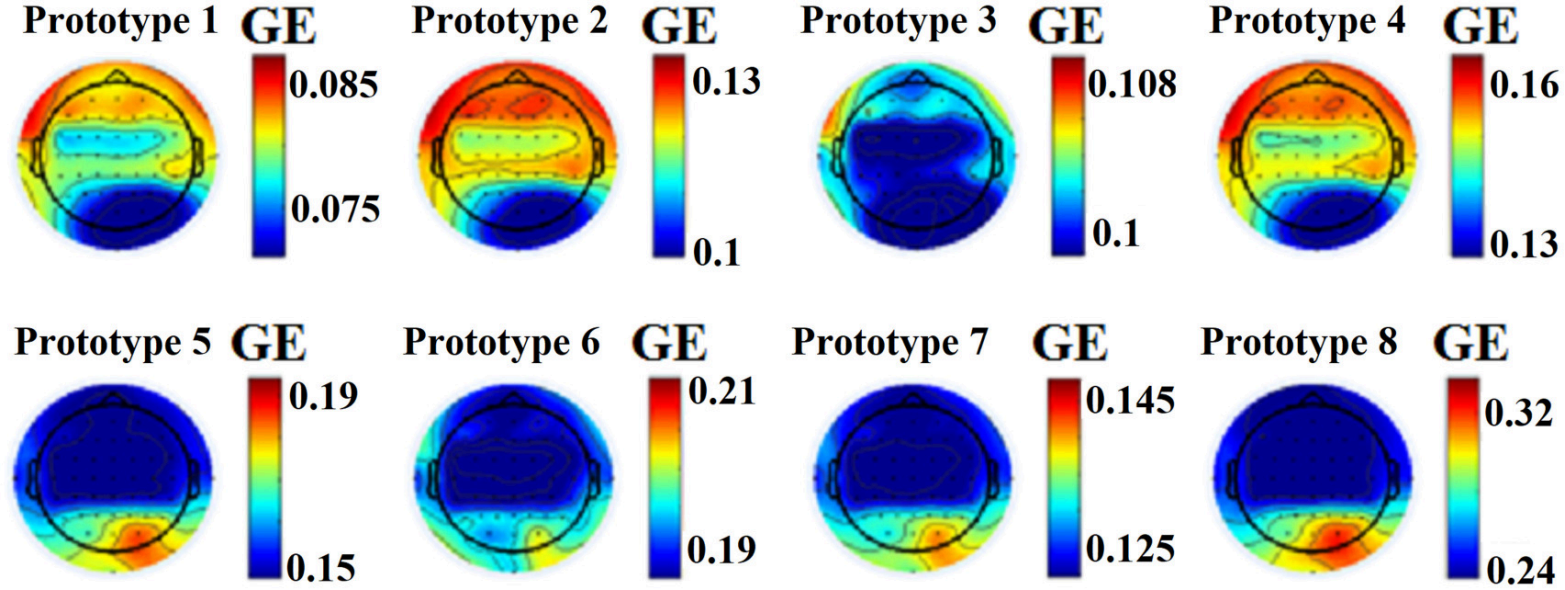
nFCμstates for eyes-open condition derived from the NNMF-VQ scheme, GE, global efficiency.

**Figure 3:**
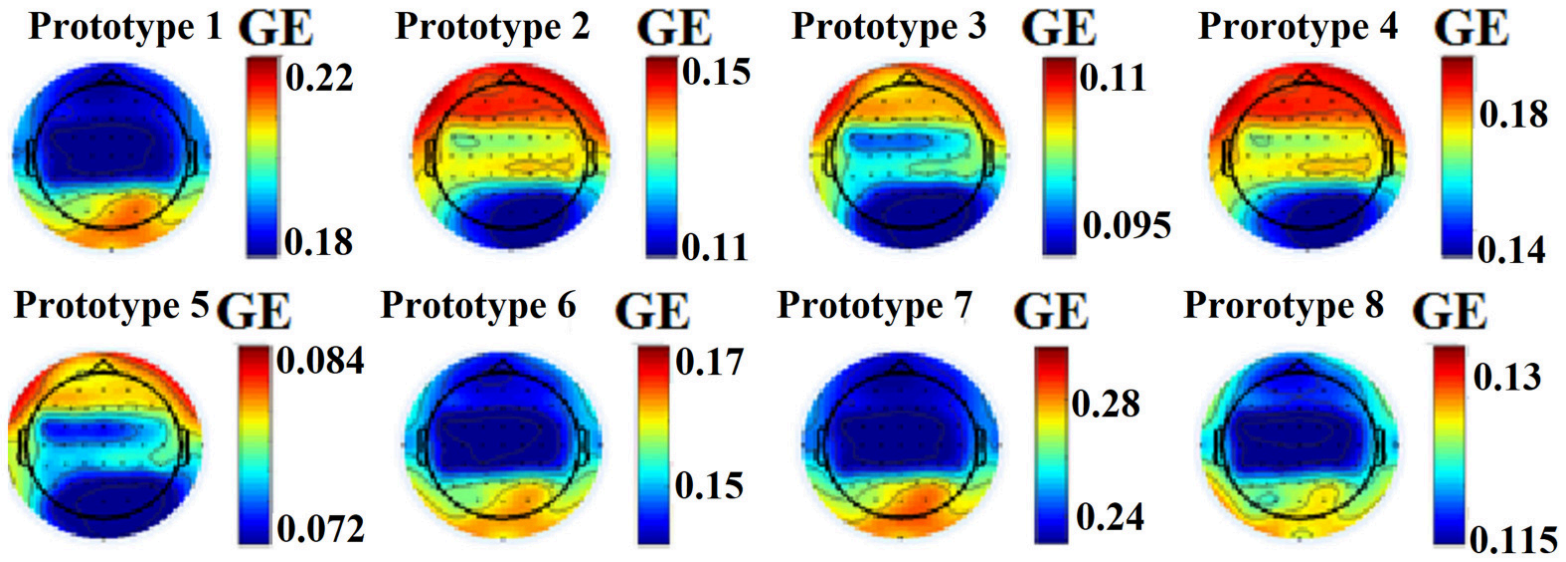
nFCμstates for eyes-closed condition derived from the NNMF-VQ scheme, GE, global efficiency.

This approach leads to eight brain metastates for eyes-open and eight for eyes-closed based on the minimization of the average distortion error (Figures 1G, 2, 3). Both Figures 2, 3 illustrates the topology of the nodal GE across the eight nFCμstates in both conditions. The nFCμstates no: 4–8 for the eyes-open condition and the nFCμstates no: 6–7 for the eyes-closed demonstrates a topological similarity over parieto-occipital sites but with different activation profile expressed via the nodal GE.

**Figure 4:**
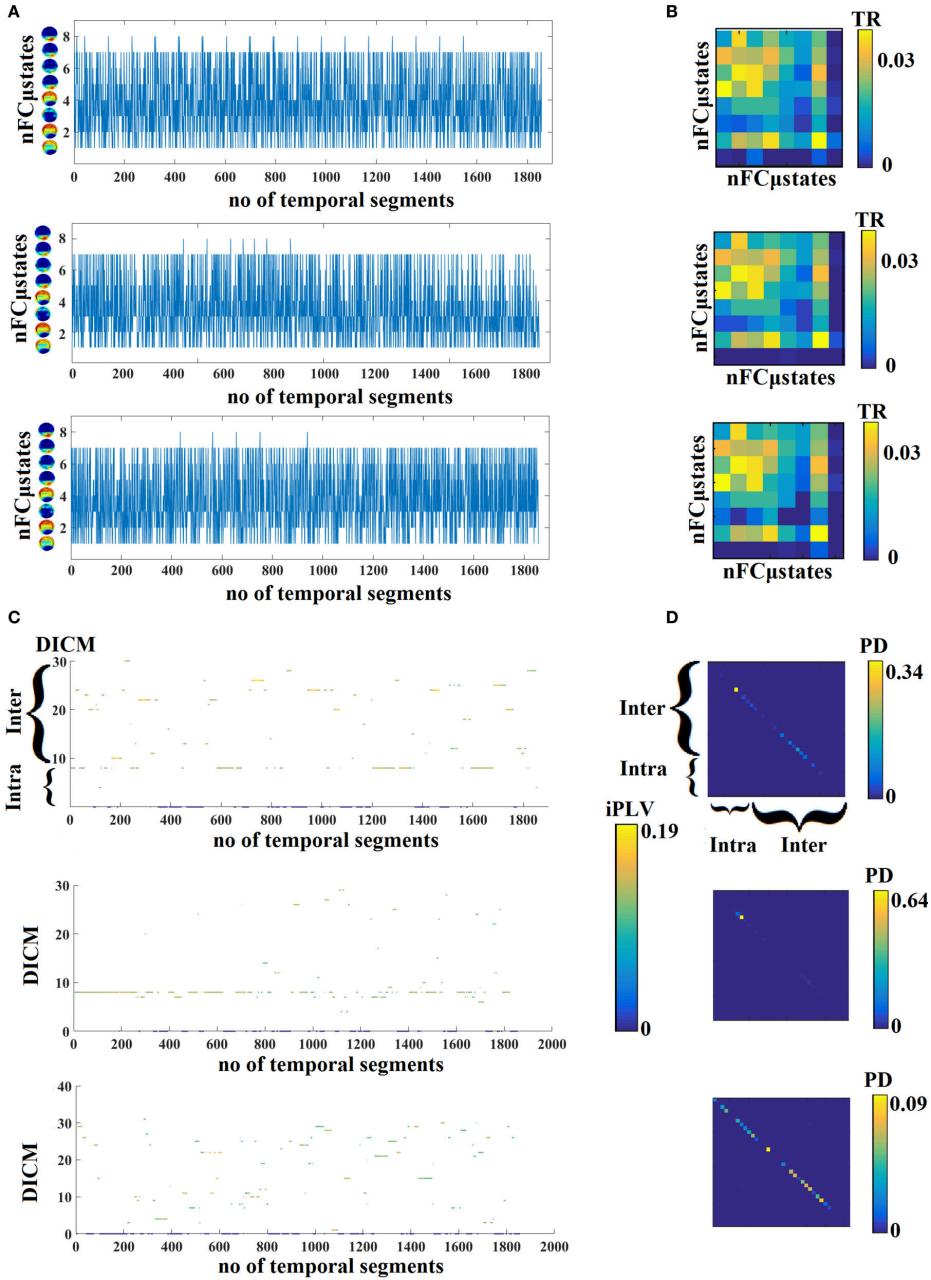
**(A)** State transition vectors for three examples (age = 18, 37, 48). Assigned states are plotted at every time point that corresponds to the center of the sliding window. **(B)** Transition matrices of the state vectors for the same example as in **(A)**. **(C)** Evolution of DICM and the related iPLV strength at every time point that corresponds to the center of the sliding window. The color refers to the iPLV strength while the y-axis refers to one of the 36 DICM. **(D)** Comodulograms of the evolved DICM from the examples in **(C)** that tabulate the probability distribution of observing each of DICM. For this particular example, we adopted the pair of F3-PO3 from the same subjects as in **(A,B)**. DICM, dominant intrinsic coupling modes; TR, transition rates; PD, probability distribution; ERT, entropy reduction rate.

In Figure 4A, we present four state transition vectors STS^NNMF-VQ^ from three subjects each one from three age groups. These STS^NNMF-VQ^ expressed the evolution of the eight nFCμstates across the experimental time. The derived transition matrix (TM) that tabulate the probability distributions of the favored transitions is demonstrated also in Figure 4A. Higher values in TM indicate preferred transitions between two states. Figure 4C demonstrates the evolution of dominant intrinsic coupling mode from pair of sensors F3-PO3 from the same subjects as in Figure 4A. The color refers to the strength of coupling namely the iPLV while the changes over the y-axis refer to the alterations of dominant intrinsic coupling modes (DICM). Across time (Figure 4C). On the right of these symbolic time series, we showed the comodulograms that tabulate the probability distribution of each of the 36 DICM for this particular pair of sensors (Figure 4D). The symbolic time series that encapsulate the DICM is a time series with values taken from 1 to 36 and it is called hereafter STS^DICM^.

### Characterization of time-varying connectivity

Once the DIFCG is formed and it is modeled via the NNMF-VQ scheme, relevant features can be extracted from the data based on the state-transition states and also on the DICM. Our approach adds in the literature also the notion of DICM which is an important characteristic of electroencephalography (Jirsa and Müller, 2013; Dimitriadis et al., 2015b, 2016a). DICM especially based on phase interactions within frequencies and phase-to-amplitude between frequencies are characteristic for EEG signal but not for fMRI. This section will describe the whole repertoire of features that were used here to build the developmental index.

### Chronnectomics

#### State transition rate

**Figure 5:**
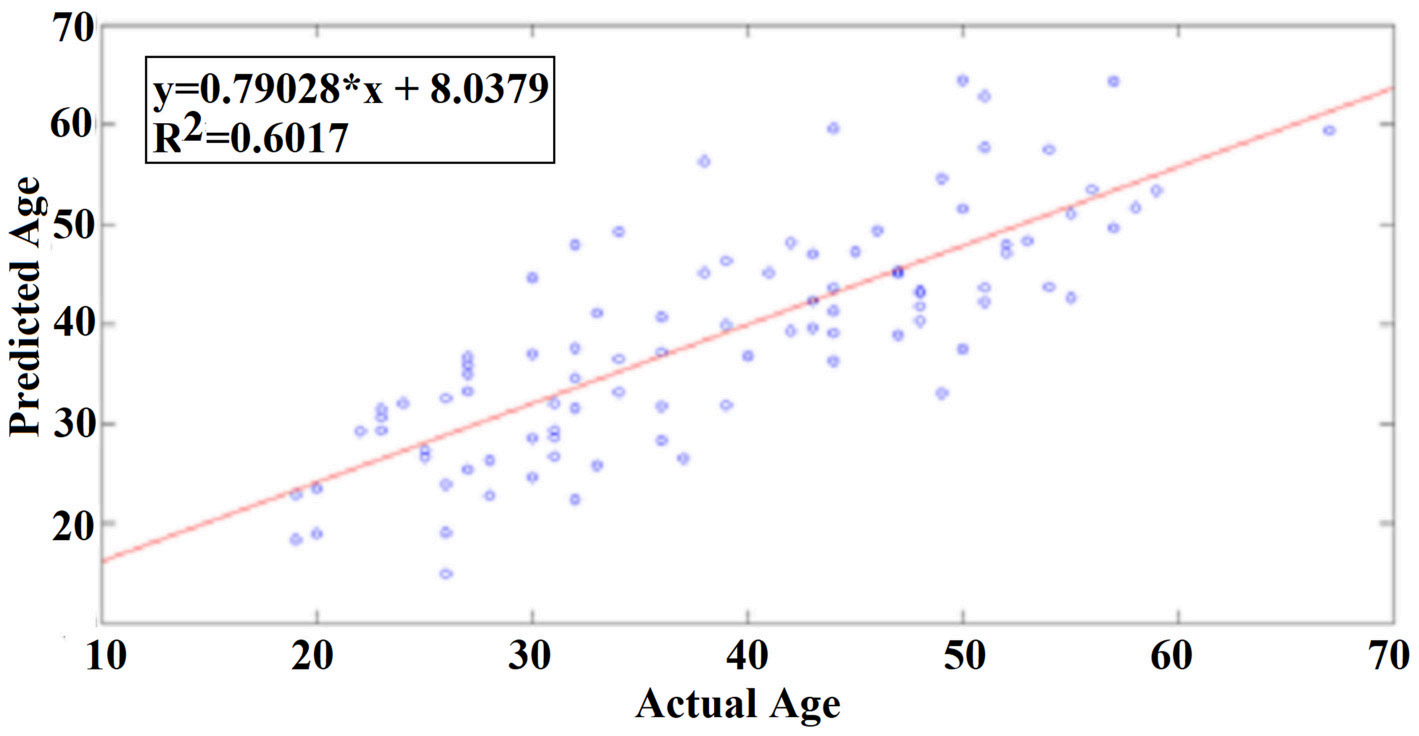
A least square regression line between the real and predicted age for eyes open.

Based on the state transition vectors STS^NNMF-VQ^ as demonstrated in Figure 5, we estimated the transition rate for every pair of states as followed:

(2) $$\begin{array}{l} TR=\frac{no\;of\;transitions}{slides-1} \end{array}$$

TR gets higher values for higher “jumps” of the brain between the derived brain states over consecutive time windows. This approach leads to one feature per participant. TR was estimated over STS^NNMF-VQ^ as demonstrated in Figure 4A.

#### Transition matrix

Complementary to TR, we estimated the transition matrix (TM) that tabulates the preferred transition of every nFCGμstates to another. TR was estimated over STS^NNMF-VQ^ (Figures 1H, 4A) and in every cell in the TM, the total number of transitions are encountered between every pair of nFCGμstates. For example, if nFCGμstates (1) transits to nFCGμstates (2) 45 times then TM _(1, 2)_ = 45 while if nFCGμstates (2) transits to nFCGμstates (1) 10 time then TM_(2, 1)_ = 10.

Finally, we transformed the transition matrix (TM) into a probability matrix by dividing the TM with the sum of totally observed transitions over all possible pairwise states. TR was also estimated between every possible pair of states leading to an 8 × 8 TR matrix and extra 64 features per subject and condition (see Figure 4B).

#### Dominant intrinsic coupling mode transition rate

Based on the 2nd DIFCG that keeps the information of the DICM per pair of sensors and across time, we estimated the transition rate for each pair of sensors. The proposed estimator can be seen as the flexibility index (FI) proposed to quantify how many times a node changes cluster assignment across experimental time (Bassett et al., 2011). The analogy of this FI for EEG is the one proposed here based on DICM and called hereafter FI^DICM^ which is defined as:

(3) $$\begin{array}{l} \text{F}{\text{I}}^{\text{DICM}}=\frac{no\;of\;transitions}{slides-1} \end{array}$$

FI^DICM^ gets higher values for higher “jumps” of preferred DICM of a pair of brain areas between consecutive time windows. This approach leads to 64 × 64 features per subject, one for every pair of EEG sensors. FI^DICM^ was estimated over STS^DICM^ as demonstrated in Figures 1I, 4C.

#### Spatio-temporal distribution of dominant intrinsic coupling modes—comodulograms

Based on the 2nd DIFCG that keeps the information of the DICM, we can tabulate in a 8 × 8 matrix the probability distribution of observing each of the 36 frequencies (8–frequencies + 28-cross-frequency pairs) across time (1,896 temporal segments) and space (64 EEG sensors). This approach leads to 36 features per subject. The matrices that keep this information are called hereafter as comodulograms. An example of these comodulograms is given in Figures 1E, 4D.

#### Complexity index

Next, a complexity index (CI) was estimated for each subject based on STS^NNMF-VQ^. CI quantifies the “richness of the language” within a symbolic sequence, and has been used in several fields, such as data compression, data mining, computational biology computational linguistics (Leve and Seebold, 2001). We adopted the complexity index based on symbolic sequences as presented in Janson et al. (2004). Low CI values describe sequences containing frequent repeated substrings that become periodic.

The magnitude of the derived CI values was evaluated according to their deviation from the maximum complexity that can be derived by random versions of the original symbolic sequence. To achieve this CI values were z-score transformed using the standard deviation of the distribution of 1,000 randomized versions of the original symbolic sequence (Dimitriadis et al., 2016b).

In addition to determining the optimal number of distinct symbols describing the original time series, the length of potentially repeated patterns of symbols (i.e., substrings) in a symbolic sequence was parameterized. Here, we fixed the parameter of the length of words that are observed from the STS^NNMF-VQ^ as length = 7. CI is estimated as the sum of distinct words detected from the symbolic sequence up to a predefined word length. The original symbolic sequence, here the STS^NNMF-VQ^ was shuffled for no = 1.000 times and the CI^Rand^ was reestimated. Then, the z-score was computed from the original CI and the mean(average)/st.d(standard deviation) of the 1.000 CI^Rand^. If the distribution of these distinct words is called symbolic patterns with dimension equals the word length, here 7, then the CI, CI^Rand^ and the z-score are defined as:

(4) $$\begin{array}{l} CI=\sum_{k=1}^{word\;length}Symbolic\;Pattern(k)\\ CI^{Rand}=\sum_{k=1}^{wordlength}Symbolic\;Pattern^{Rand}(k)\\ Z(CI)=\frac{CI-mean(CI^{Rand})}{std(CI^{Rand})} \end{array}$$

In a simplified example of the symbolic sequence {1 2 1 2 1 2 1 2 1 2}, the distinct words (symbolic patterns) of length up to 2 are the {1,2,12,21} and the CI = 4. In a second symbolic sequence of {1 1 1 1 1 1 1 1 1 1}, the distinct words (symbolic patterns) of length up to 2 is only the {1,11} and the CI = 2. To further understand the notion of CI, a third example of symbolic sequence is provided {1 2 2 1 2 2 1 2 1 1}. The symbolic patterns based on words of length = 2 is consisted of 6 distinct words {1,2,12,22,21,11}. The CI equals to 6.

STS^NNMF-VQ^ describes the deterministic nature of the human brain where hundreds of time-instant functional connectivity graphs can be described by a small repertoire of nFCGμstates. Complexity index can express how repeatable are the patterns of brain connectivity across experimental time. We assume that the CI of the human brain dynamics will be higher for younger subjects compared to middle-aged group which means that the human brain becomes more deterministic across the age.

The whole approach leads to a single feature per subject. For further details of the complexity index, an interested reader could see Section 5 in Supplementary Material and also the original article (Janson et al., 2004).

#### Entropy of Markov trajectories

By adopting the aforementioned NNMF-VQ scheme, we modeled the IDFCG into distinct nFCμstates. Under this scheme, we transformed a dynamic network into a symbolic time series that preserve the information of the derived nFCμstates at every time point (see Figure 4A). This time series can be seen as a finite-state machine. A Markovian chain modeling can be reasonable derived from the symbolic time series STS^NNMF-VQ^. This type of modeling emphasizes the transition between the nFCμstates.

Aiming to detect features that can be valuable for the building of a chronnectomic maturation index based on EEG, we resort to a technique that quantifies the entropy of Markov trajectories which are routes starting from a particular microstate and ending to another. Our assumption based on the notion of a less flexible brain, less degrees of freedom through the age. This less flexibility can be estimated as a reduction of the entropy of specific Markovian trajectories which are considered as a finite irreducible Markovian chain with empirical transition matrix P which is derived from the STS^NNMF-VQ^. The entropy is defined as:

(5) $$\begin{array}{l} H\left(X\right)=-\underset{i,j}{\sum }\mu_{i}P_{ij}\log P_{ij} \end{array}$$

where μ is a n equilibrium distribution of the Markov chain as below:

(6) $$\begin{array}{l} \mu_{j}=\underset{i}{\sum }\mu_{i}P_{ij}\; \end{array}$$

For an irreducible Markov chain, the entropy *H*_*ii*_ of a random trajectory from state *i* back to state *j* is given by the following equation

(7) $$H_{ii}=\frac{H(X)}{\mu_{i}}$$

where μ_*i*_ is the stationary probability for state *i* and *H*(*X*) is the entropy rate given in Equation 5.

If *P*_*ij*_ is the transition matrix of an irreducible finite state Markovian chain, then the entropy *H*_*ij*_ of the trajectory from *i* to *j* is defined as:

(8) $$\begin{array}{l} \;H=K-K\prime +H_{\Delta }\\ \text{ where} \end{array}$$

(9) $$\begin{array}{l} K={\left(I-P+A\right)}^{-1}\left(H^{*}-H_{\Delta }\right) \end{array}$$

(10) $$\begin{array}{l} K\prime_{ij}=K_{jj} \end{array}$$

(11) $$\begin{array}{l} A_{ij}=\mu_{i} \end{array}$$

(12) $$\begin{array}{l} H_{ij}^{*}=H(P_{i})\\ \text{ for all }i,j\text{ and} \end{array}$$

(13) $$\begin{array}{l} {\left(H_{\Delta }\right)}_{ij}=\left\{\begin{array}{l} H\left(X\right)/\mu_{i},i=j\\ 0,i\neq j \end{array}\right\} \end{array}$$

In the present study, we employ the finite state irreducible Markov chains modeling scheme focusing on the observed trajectories *T*_*ij*_. A trajectory *t*_*ij*_ϵ*T*_*ij*_ from state *i* to state *j* is a path with initial state *i*, final state *j* without intervening state *j*.

For further details see the seminar work of Ekroot and Cover (1993).

The whole approach leads to an 8 × 8 matrix per subject that tabulates the entropy of Markovian trajectories called entropy reduction rate (ERT). Those 64 features will be added to the aforementioned features.

#### Sample entropy

We created a strength time series by summing the weights of the survived functional connections (after surrogate analysis and topological filtering) of each time instant IFCG. This procedure produces a strength time series of 1,856 samples length. Afterward, we estimated the sample entropy of this time series as described here (Richman and Moorman, 2000).

## Feature selection and prediction of maturation

### Feature selection

The whole repertoire of features proposed here leads to a total number of 1 (TR) + 8 × 8 (TM) + 64 × 64 (FI) + 36 (comodulograms) + 1 (Flexibility Index) + 8 × 8 (entropy of Markovian trajectories) + 1 (Sample Entropy) = 4.262 features per subject. For that reason, we should apply an appropriate feature selection approach for both tasks: (a) prediction of age and (b) discrimination of young vs. middle age adults.

### Prediction of age

We estimated the distance correlation of 4262 features across subjects leading to a 4.262 × 4.262 matrix with positive weights. Distance correlation *distcor* ranges within [0, 1] in contrast to the correlation coefficient and can capture non-linear relationships (Szekely and Rizzo, 2014). Afterwards, we applied a graph clustering algorithm (Massimiliano and Pelillo, 2007) to extract clusters of features that are highly correlated within the cluster and low correlated between clusters. Our analysis revealed 25 clusters for the eyes-open condition and 43 for the eyes-closed. Finally, we extracted one feature per cluster with the criterion of high correlation with the age across the cohort. This procedure leads to 25 features for eyes-open and 43 features for eyes-closed.

As an appropriate method for build a model for predicted the age based on the selected features is the extension of SVM which is called SVM regression (SVR). Drucker et al. (1997) extended SVM to include also the regression approach for continuous real valued predictions. SVR retains the basic features of SVM classifier but the key difference is that in SVM classification a penalty exists for misclassified data points whereas in SVR a penalty is used for data points far from the regression line in a high dimensional space (Dosenbach et al., 2010).

### Classification of young vs. middle-age adults

For the binary classification of the two groups, we followed a different feature selection approach. By adopting the Laplacian Score (He et al., 2005), we estimated the weights of each feature that quantifies the discriminative power of each feature. On this procedure, we used the labels of 1 for young group and 2 for middle-age group. In order to apply a threshold, we re-estimated the LS of the features by shuffling the labels [1, 2] across the subject. We repeated the procedure 200 times and afterward we estimated the mean + 2.5 std (standard deviations) from the 1.000 × 4.263 LS weights. This thresholding criterion was applied to the original LS weights leading to the selection of 109 features.

As a proper classifier for the classification of age groups, we used the famous extreme learning machine (ELM). ELMs appeared as a proper choice due to its ability to manipulate difficult tasks without an extensive training set up (Huang et al., 2006). ELM are feedforward artificial neural networks with a single layer of hidden nodes where the weights that connect the input to the hidden nodes are randomly assigned and never updated (Huang, 2015).

## Results

### Prediction of age

We followed a leave-one out cross-validation scheme (LOOCV) in order to predict the age of a subject. This means that we trained the classifier with the selected features from *N* − 1 where *N* denotes the number of subjects and we predicted the age of the *N*th. Repeated the whole approach *N* times, we predicted the individual age of every subject in the cohort. For the purpose of the prediction of age, we adopted the regression version of the Support Vector Machine, the Support Vector Regressor (SVR).

**Figure 6:**
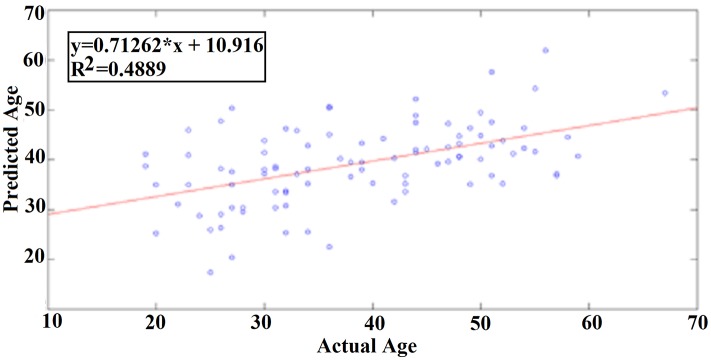
A least square regression line between the real and predicted age for eyes-closed.

Our results revealed a very high prediction of age for eyes-open (*R*^2^ = 0.60; y = 0.79x + 8.03) and lower for eyes-closed (*R*^2^ = 0.48; y = 0.71x + 10.91) where y is a linear regression line applied to the (x, y) points with x being the true age of the subject and y the predicted age (Figures 5, 6).

**Figure 7:**
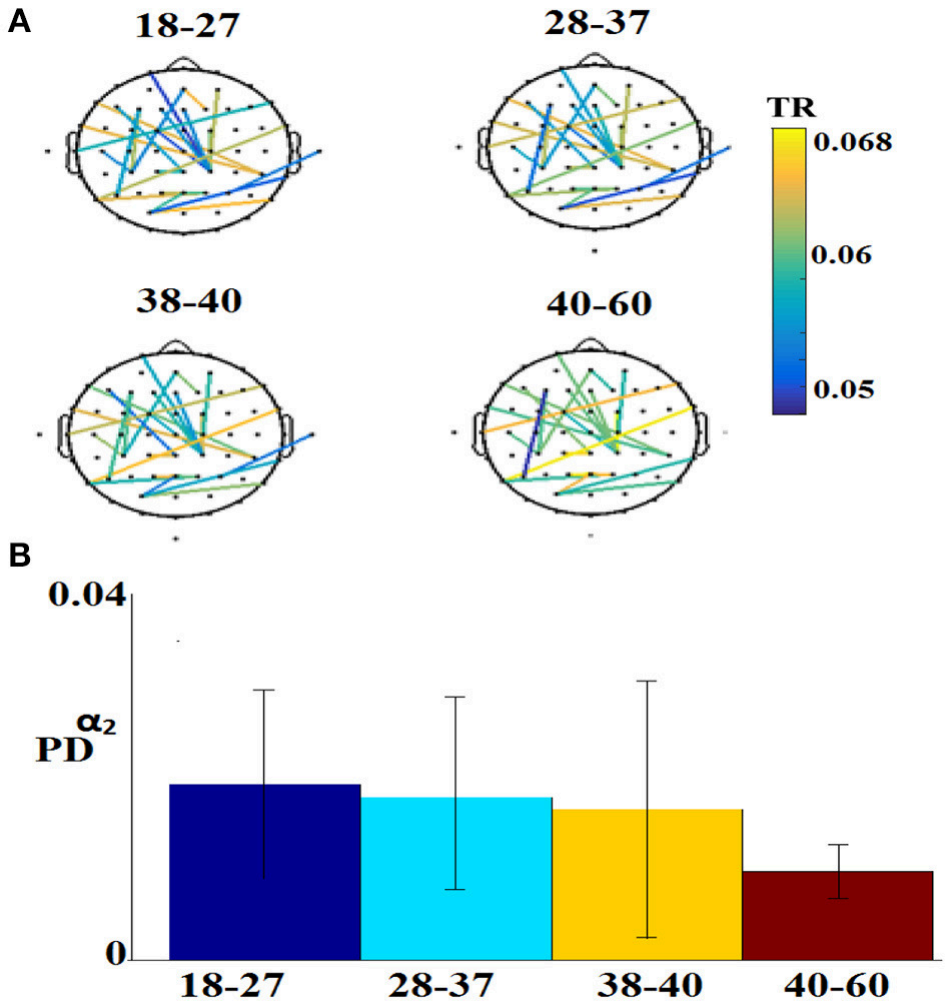
Demonstration of features for the regression problem. **(A)** Nineteen features referred to FI per pair of sensors and **(B)** for the PD for α_2_. TR, transition rates, PD, probability distribution.

Figure 7 illustrates the 25 selected features for the regression problem of prediction of actual age based on eyes-open. The 25 selected features referred to 24 FI per pair of sensors (Figure 7A) and 1 for the PD for α_2_ (Figure 7B).

### Classification of young adults vs. middle age group

Using extreme learning machine (ELM classifier and a LOOCV), we succeeded to correctly classify young vs. middle-age group with 97% accuracy in eyes-open and 87.2% for eyes-closed (Table 1).

**Table 1:** Accuracy, sensitivity, and specificity for the two-class classification problem for the training dataset.

	**Accuracy (%)**	**Sensitivity (%)**	**Specificity (%)**
Eyes-Open	97	100	95
Eyes-Closed	87.23	88.24	86.05

**Figure 8:**
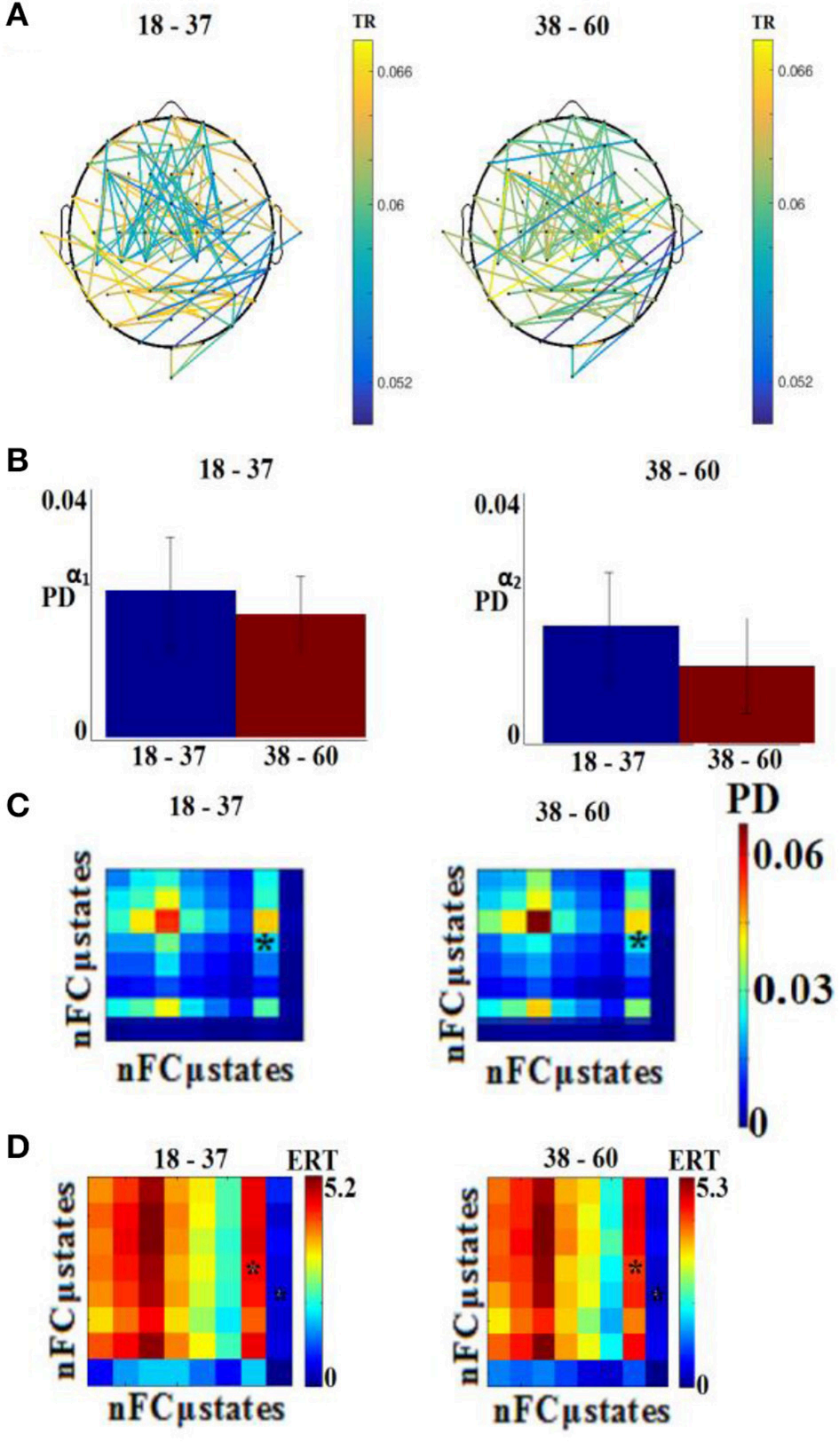
Demonstration of features for the classification problem. **(A)** One hundred and eight features referred to FI per pair of sensors, **(B)** for the PD for α_1_ and α_2_, **(C)** from the transition matrix between state 4 and 7 and **(D)** 2 from the entropy reduction rate on between {4,7} and {8,5} pairs of states (^*^ denotes statistically significant features detected via Wilcoxon Rank-Sum Test, *p* < 0.001). TR, transition rates; PD, probability distribution; ERT, entropy reduction rate.

Figure 8 illustrates the distribution of selected features for the classification problem across the different sub-groups of features. Our feature selection analysis revealed 108 features referred to FI per pair of sensors (Figure 8A), 2 based on the PD for α_1_ and α_2_ (Figure 8B), 1 from the transition matrix between state 4 and 7 (Figure 8C), and 2 from the entropy reduction rate on between {4,7} and {8,5} pairs of states (Figure 8D). This makes a total of 103 features from a total of 4.262 initial pool of features.

### External validation

**Figure 9:**
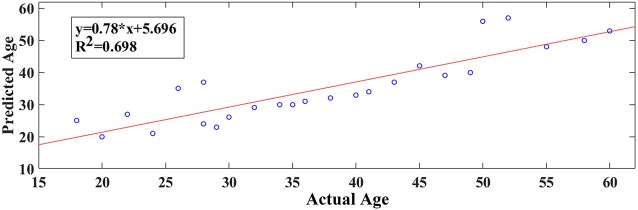
A least square regression line between the real and predicted age for eyes open for the external validation dataset.

**Figure 10:**
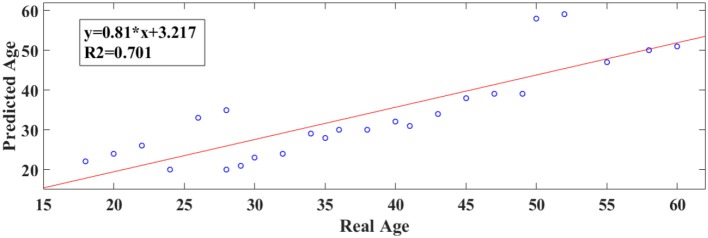
A least square regression line between the real and predicted age for eyes closed for the external validation dataset.

Following an external validation of the whole approach in a cohort of 100 subjects, we performed reasonably well in continuous age prediction for eyes-open condition (*R*^2^ = 069, y = 0.78x + 5.696) and eyes-closed condition (*R*^2^ = 070, y = 0.81x + 3.217). The ELM classifier discriminated between young (18–37) and midde-aged subjects (40–60) with 98% accuracy (*p* < 10^−7^) for eyes-open and with 97% accuracy (*p* < 10^−9^) for eyes-closed (Table 2). Figure 9 illustrates the prediction for eyes-open and Figure 10 the prediction of eyes-closed.

**Table 2:** Accuracy, sensitivity, and specificity for the two-class classification problem for the second testing dataset.

	**Accuracy (%)**	**Sensitivity (%)**	**Specificity (%)**
Eyes-Open	98	100	97
Eyes-Closed	85.48	86.78	85.83

## Discussion

In the present study, we examined a novel algorithmic approach of modeling dynamic functional connectivity graphs via NNMF-VQ symbolization approach. Additionally, we demonstrated how to integrate into a single dynamic functional connectivity graph (IDFCG) both the weight and also the DICM of each connection (Figures 1B,C). This novel approach first presented here demands novel statistical framework in order to extract the DICM among intra and inter-frequency coupling modes (Statistical Filtering). Afterward, we applied to DIFCG, a novel data-driven topological filtering algorithm with main aim to increase the information flow within a network under the constrained of the overall cost of the survived connections. This approach overcomes all the existing arbitrary approaches that bias any observations in brain networks. Here, we used a data-driven topological filtering scheme which is called Orthogonal Minimal Spanning Trees (OMST; Dimitriadis et al., 2017). To demonstrate the effectiveness of the OMST to the prediction of age and the classification of age groups, we repeated the whole analysis using three famous arbitrary filtering schemes. The results clearly proved the significance of the topological filtering scheme to the overall analysis (See Section 6 in Supplementary Material). The most trivial arbitrary thresholding schemes are the absolute threshold, the density of the strongest connections and mean degree of a network. After both statistical and topological filtering applied to IDFCG, we applied a data-driven NNMF-VQ scheme to uncover nFCGμstates at each condition with minimal reconstruction error. Afterward, we presented novel chronnectomics that can be extracted from both the evolution of nFCGμstates and also the evolution of DICM.

The main highlights of our research can be summarized as follow:

1. We presented for the first time a CBMI based on EEG modality.
2. Our analysis focused on mining integrated dynamic functional connectivity networks under the notion of DICM and connectivity microstates analysis.
3. We demonstrated a whole repertoire of complementary features integrated in time, space and in both dimensions.
4. These features potentially succeeded to predict the individual maturation age and to discriminate with high precision young vs. middle-aged subjects.
5. Finally, we replicated our findings in a second external dataset.

The whole repertoire of nFCGμstates in both tasks illustrated an activity in frontal, parieto-occipital, and also in temporal brain areas bilaterally. Frontal activity is directly linked to various cognitive tasks requiring attention or working memory (Burgess and Gruzelier, 1997) while frontal and parietal activity has been associated with working memory functions and short-term memory (Calhoun and Adali, 2012).

The chronnectomics that were presented here are the entropy reduction rate (ERT) based on the Markovian Chain of the symbolic time series that keeps the evolution of nFCGμstates across experimental time. Additionally, transition rate (TR) of these nFCGμstates is a novel estimator that quantifies the preferred transition between specific nFCGμstates (Calhoun and Adali, 2012). Similarly, we proposed flexibility index (FI), a novel measure that quantifies how many times a pair of EEG sensors alters favorable DICM. FI can be seen as how flexible is a brain to alter its DICM across time. Finally, the probability distribution of each DICM across spatio-temporal domain can be seen as a novel characteristic fingerprinting of the preferred intra or cross-frequency coupling across time and brain network.

The whole methodology was presented to solve for the first time in EEG analysis, a highly active research area to define an objective maturation index. Vergun et al. (2013) studied rs-fc-fMRI by combing brain networks and machine learning techniques. Recently, Qin et al. (2015) revealed complementary information of resting-state based on fMRI by adopting a dynamic functional connectivity graph. He showed that the variability of the strength between specific brain areas reduced with the increment of age (Qin et al., 2015). Apart from fMRI, an EEG was missing in the literature based on resting-state.

Here, we defined a novel multi-parametric chronnectomic maturation index (CMI) based on EEG resting-state activity. We performed reasonably well in continuous age prediction for eyes-open condition (*R*^2^ = 0.60) and eyes-closed condition (*R*^2^ = 0.48). In an external second set of 25 subjects, we performed higher prediction for eyes-open (*R*^2^ = 0.69) and eyes-closed (*R*^2^ = 0.70). The difference between the training dataset and the external in terms of prediction accuracy could be interpreted as a more objective CMI compared to the chronological age and more directly connected to the human maturation age. Further studies should explore the link of the whole approach to a unique phenotype for each subject.

Resting-state can be seen as a baseline of the human brain activity. The activity of every subsystem like visual, auditory, attention, etc., during targeted tasks can be predicted well by their activity at resting-state. A recent study based on rs-fMRI succeeded to link the activity at resting-state with the activity at specific locations in tasks that activate specific well-known subsystems (Cole et al., 2016). The novel proposed FI based on DICM can be seen as the reflex of every part of the brain to adjust to external stimuli. The more flexible is the brain, the more ready to react and this can be seen at resting-state. It would be interesting in the future to link FI at resting-state with task-related FI between specific subsystems (Buzsaki, 2010; Buzsaki et al., 2013). Flexibility index was the most significant feature in both building a chronnectomic brain aged index (CBAI) and also to the high discrimination of the two age groups in both datasets.

We are working in the era of a great evolution in the neuroscience community when multimodal neuroimaging techniques applied to both daily clinical practice and also to neuroimaging centers are combined with complex network analysis. It is more than evident that neuroscience is still looking to find a way to reveal a relationship between behavioral responses and brain activity. Especially, for dynamic functional connectivity there is not an easy way to build up such a connection. Recently however, neuroscientists combined a pharmacological intervention with novel techniques from dynamic network neuroscience applied to fMRI to identify alterations in the dynamic reconfiguration of brain networks related to schizophrenia genetic risk and NMDA receptor hypofunction (Braun et al., 2016). They quantified “network flexibility,” as a measure of the dynamic reconfiguration of the community structure of time-variant brain networks during working memory performance. This flexibility quantifies how many times a node/ROI changes cluster assignment over experimental time. The whole approach based on the weighted correlated dynamic brain networks. Here, we defined a novel flexibility index based on the rate of dynamic reconfiguration of the dominant coupling modes across the experimental time.

The disadvantage of fMRI is that it cannot give information regarding the amplitude/phase and/or frequency profile of dynamic connectivity (Engel et al., 2013; Dimitriadis et al., 2016a,c,d). EEG and also MEG can give us information regarding the type of interactions taking into consideration simultaneously the amplitude-phase and the frequency profile of each interactions (phase-to-phase, amplitude-to-amplitude, cross-frequency coupling).

The present study is the very first in the literature that takes into consideration all of these parameters linked to the nature of the imaging modality (EEG). The presented methodology shows how to construct a single dynamic functional connectivity graph per subject that encapsulates both the strength but also the nature of the dominant intrinsic coupling mode and is of paramount importance for the neuroscience community. Here, in analogy of the fMRI-based flexibility index^6^, we designed a flexibility index that quantifies how many times across temporal segments at resting-state, two brain areas alter their preferred type of interaction. The higher the frequency of their alterations, the higher the flexibility index and the more flexible is the brain in global. We observed that the most informative feature for the prediction of age and discrimination of the two age groups was the Flexibility Index for a subset of pairs of EEG sensors.

## Conclusions

Our own work supports that resting state EEG data contains adequate information to predict individual actual age and also to predict age-group subjects. As the amount of free available EEG datasets will be increased, data-driven techniques applied to dynamic functional connectivity graph should be adopted. With the incorporation on the whole analysis of appropriate features, feature selection, and machine learning algorithms will extract meaningful information that will improve human clinical diagnoses and the overall efficacy.

## Author contributions

Conception of the research, methods and design, critical revision of the manuscript, and drafting the manuscript: SD. Data analysis: SD and CS. Every author read and approved the final version of the manuscript.

### Conflict of interest statement

The authors declare that the research was conducted in the absence of any commercial or financial relationships that could be construed as a potential conflict of interest.

## Footnotes

^1^https://physionet.org/pn4/eegmmidb/

## Supplementary material

The dataset is a free available EEH study that can be downloaded from here: https://physionet.org/pn4/eegmmidb/.
