# Supplementary material for: Mining Time-Resolved Functional Brain Graphs to an EEG-Based Chronnectomic Brain Aged Index (CBAI)
Source: Front Hum Neurosci. 2017 Sep 7;11:423. doi: 10.3389/fnhum.2017.00423 (PMC5594081; doi:10.3389/fnhum.2017.00423)
Supplement: Supplementary file 1 [file DataSheet1.DOCX]

**Supplementary Material**

**Mining Time-Resolved Functional Brain Graphs to an EEG-based Chronnectomic Brain Aged Index (BAI)**

S. I. Dimitriadis^1-4*^, Christos I. Salis^5^

^1^ Institute of Psychological Medicine and Clinical Neurosciences, Cardiff University School of Medicine, Cardiff, United Kingdom

^2^ Cardiff University Brain Research Imaging Center (CUBRIC), School of Psychology, Cardiff University, Cardiff, United Kingdom

^3^ School of Psychology, Cardiff University School of Medicine, Cardiff, United Kingdom

^4^ Neuroinformatics Group, (CUBRIC), School of Psychology, Cardiff University, Cardiff, United Kingdom

^5^ Department of Informatics and Telecommunications Engineering, University of Western Macedonia, Kozani, Greece

**Contents:**

**Section 1 .. Intra-Frequency Connectivity Estimator ……………p.4**

**Section 2 ..CFC metric computation ……………………………..p.4**

**Section 3 ..Surrogate Data Analysis of *iPLV* Estimates –**

**Statistical Filtering of Brain Networks …………………………..p.7**

**Section 4..A data-driven Topological Filtering Scheme based**

**on Orthogonal Minimal Spanning Trees ……………………….p.8**

**Section 5. Complexity of individual time series as symbolic sequences…………………………………………………………. p.12**

**Section 6. The importance of a data-driven**

**topological filtering scheme – the OMST approach……………p.13**

**Section 1. Intra-Frequency Connectivity Estimator**

Among the available connectivity estimators, we adopted the one based on the imaginary part of phase-locking value (iPLV) (Lachaux *et al.*, 1999) and adjusted properly so as to extract time-resolved profiles of intra-frequency coupling from EEG multichannel recordings at resting state. The original PLV is defined as follows:


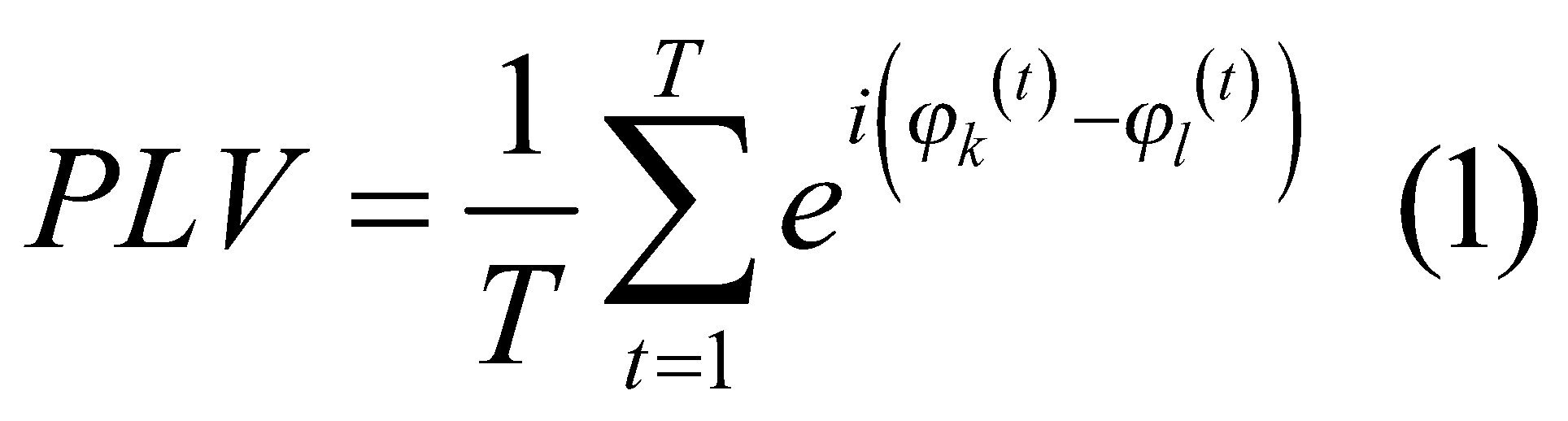


where *k*, *l* denote a pair of EEG sensors and the imaginary part of PLV is equal to:


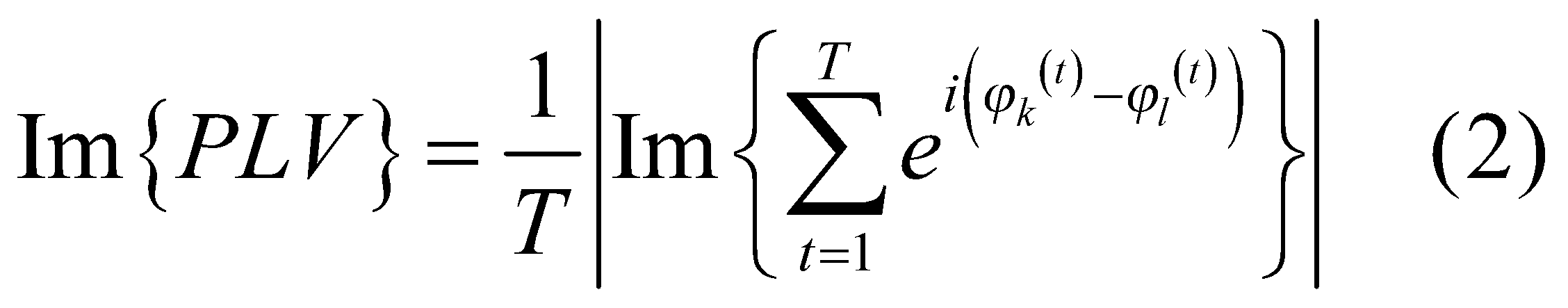


The imaginary part of PLV (iPLV) investigates intra-frequency interactions without putative contributions from volume conductance. In general, the iPLV is mainly sensitive to non-zero-phase lags and for that reason is resistant to instantaneous self-interactions from volume conductance (Nolte, 2004). In contrast, it could be sensitive to phase changes that not necessarily imply a PLV oriented coupling.

**Section 2. CFC metric computation**

- - 1. CFC estimates the strength of pairwise interactions and identifies the prominent interacting pair of frequencies, both between and within sensors (Canolty, Knight, 2010 ; Buzsáki , 2010 ; Buzsáki *et al.*, 2013). Among available CFC descriptors, phase-amplitude coupling (PAC), which relies on phase coherence, is the one most commonly encountered in research (Voytek *et al.*, 2010). The PAC algorithm as adapted to continuous MEG multichannel recordings is described below.
    2. Τhe more general case of CFC (i.e., between-sensor coupling) is described here—within-sensor CFC is derived by collapsing the two sensor indices to a common index. Let *x*(*i*_sensor_, *t*), be the MEG activity recorder at the *i*_sensor_-th site, and *t*=1, 2,.... *T* the successive time points. Given two frequency-limited signals *x*(*i*_sensor_, *t*) and *x*(*j*_sensor_, *t*), cross-frequency coupling is estimated by allowing the phase of the lower frequency (LF) oscillations to modulate the amplitude of the higher frequency (HF) oscillations. The complex analytic representations of each signal *Z*_LF_(*t*) and *Z*_HF_(*t*) are derived via the Hilbert transform (*HT*[.]).
    3.
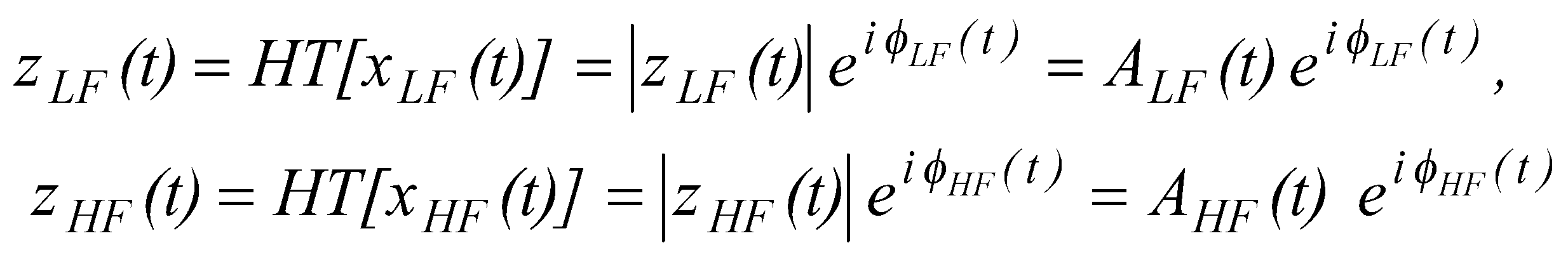
  (1)

Next, the envelope of the higher-frequency oscillations *A*_HF_(t) is bandpass-filtered within the range of LF oscillations and the resulting signal is submitted to an additional Hilbert transform to derive its phase dynamics component φ'(t)


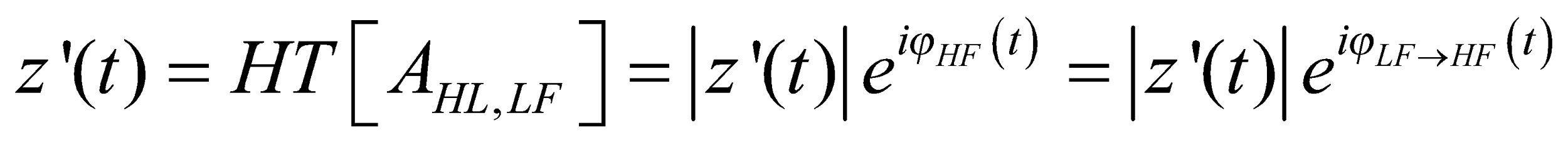
     (2)

which expresses the modulation of the amplitude of HF-oscillations by the phase of the LF-oscillations. Phase consistency between the two time series was measured by means of both the original definition (Lachaux *et al.*, 1999) and the imaginary portion of PLV, as synchronization indexes to quantify the strength of PAC. The original PLV is defined as follows:


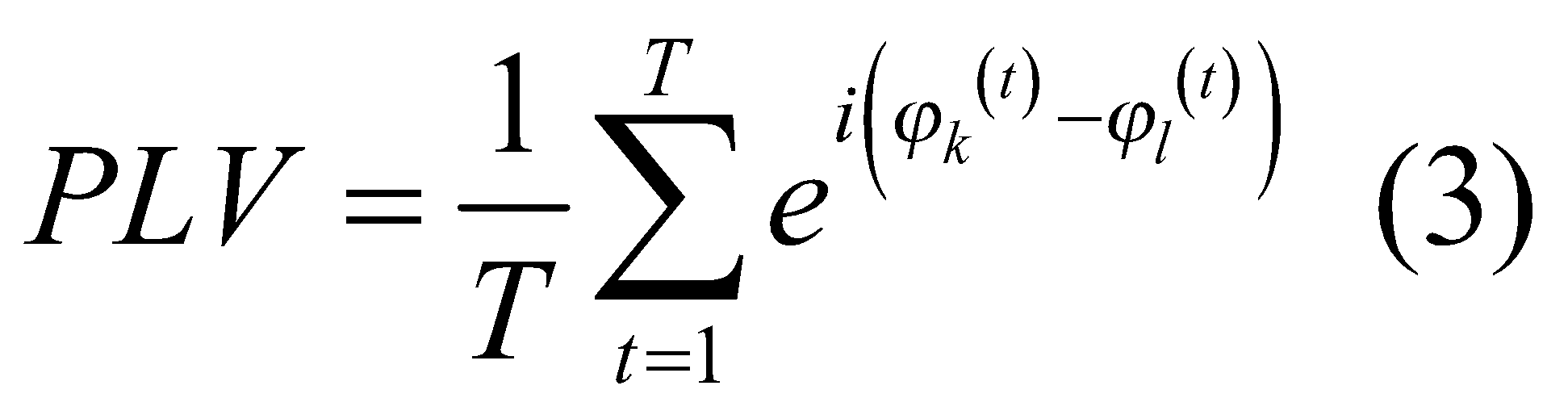


and the imaginary part of PLV as follows:


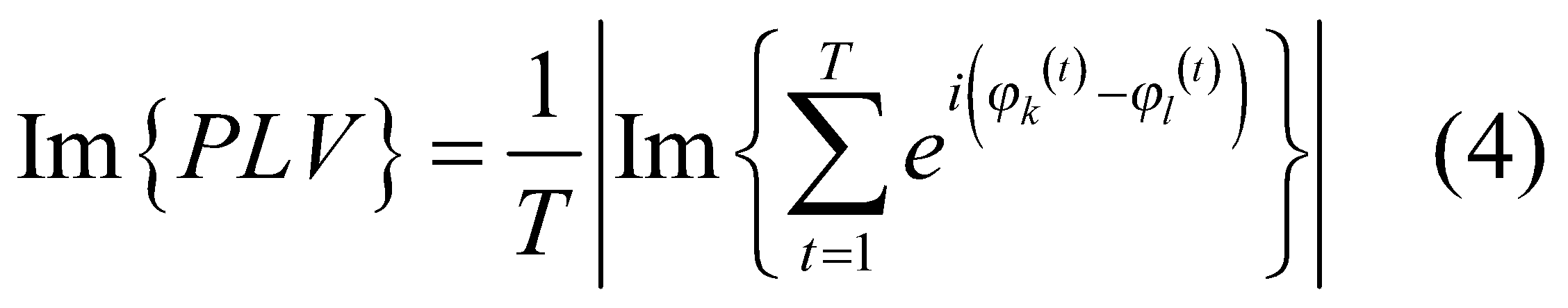


The imaginary portion of PLV is considered to be less susceptible to volume conduction effects in assessing CFC interactions. While the imaginary part of PLV is not affected by volume conduction effects, it could be sensitive to changes in the angle between two signals, which not necessarily imply a PLV change. In general, the imaginary portion of PLV is only sensitive to non-zero-phase lags and is thus resistant to instantaneous self-interactions associated with volume conductance (Lachaux *et al.*, 1999).

Figure S1, demonstrates the previous algorithmic steps using a pair of sensors located over left and right frontal areas from a sliding window from one of the NI subjects. PAC interactions are examined, between LF oscillatory activations corresponding to δ brain rhythm and HF activations corresponding to β_1_ rhythm. The original signals are shown in S1a. The HF version of signal x_1_(t) with its envelope is depicted in S.1b. S1d shows the low-pass filtered (within δ frequency range) version of the previous envelope (i.e. the A_β1,θ_(t) signal). The saw-like trace corresponds to its instantaneous phases φ'_β1_(t). On the other hand, the LF version of the signal x_2_(t) is depicted in S.1c, along with the trace of the corresponding instantaneous phases φ_θ_(t). The φ_θ_(t) and φ'_β1_(t) traces have been plot aligned in S.1e, so as to form the instantaneous phase differences as shown in S.1f. It is this sequence of phase-differences Δφ(t) that enters in equation (1) and will be ''integrated'' across time via averaging the corresponding directional vectors e^iΔφ(t)^ in the complex domain. It is important that the length T of this sequence has to be long enough, so as the iPLV index to provide a reliable estimation of PAC. We employed this PAC CFC estimator also in previous studies (Dimitriadis et al., 2015,2016a,b,2017).


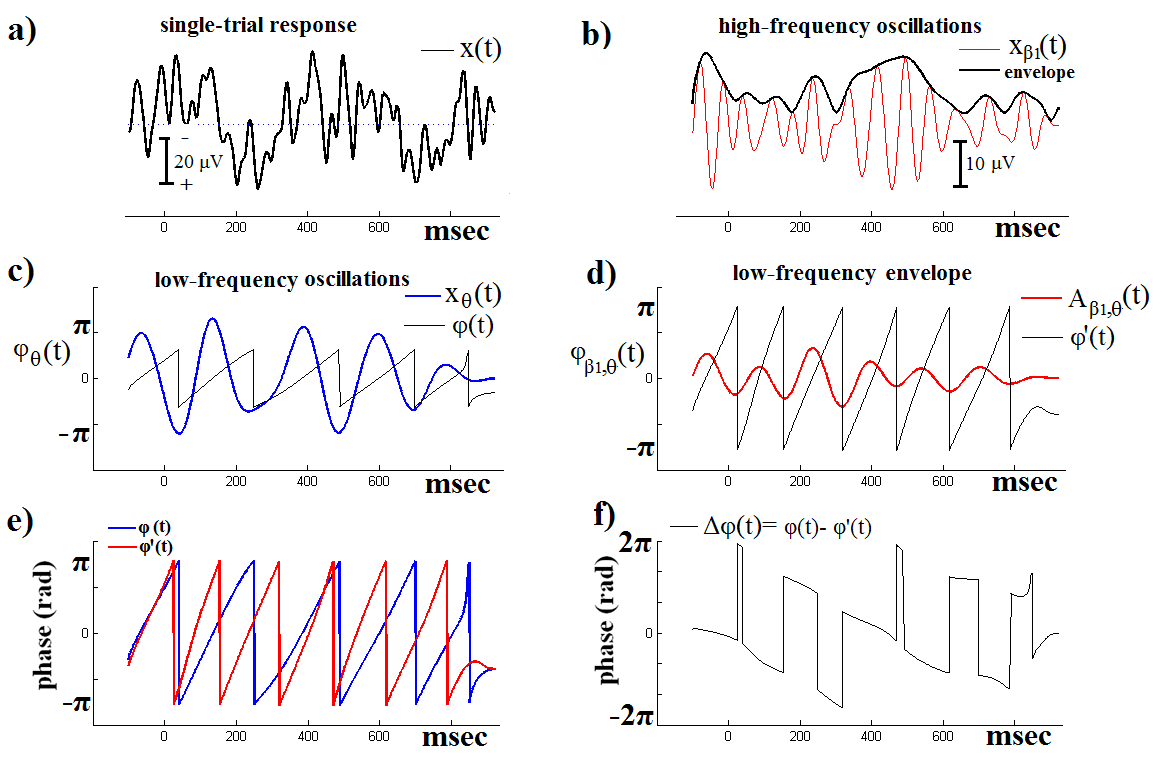


**S1. The algorithmic steps for PAC estimation.** Using the first single-trial signal a), from the cognitive responses of a control subject, we demonstrate the detection of coupling between θ and β_1_ rhythm. The time series of instantaneous phases from the low-frequency rhythm and its ‘projection’ on the amplitude dynamics of the high-frequency rhythm are presented simultaneously in e). The latency depended differences f), will be used in estimating the phase-locking that will reflect the PAC-interaction between the two involved brain rhythms.

**Section 3.Surrogate Data Analysis of *iPLV* Estimates – Statistical Filtering of Brain Networks**

To identify significant iPLV-interactions which were estimated for every pair of frequencies within and between all 64 sensors, and at each successive sliding window (i.e. temporal segment), we employed a surrogate data analysis (Theiler *et al.*, 1992). Accordingly, we could determine (a) if a given iPLV value differed from what would be expected by chance alone, and (b) if a non-zero iPLV corresponded to nonspurious

coupling. For every temporal segment, sensor-pair and frequency, we tested the null hypothesis H0: ''the observed iPLV value comes from the same distribution as the distribution of surrogate iPLV-values''. One thousand surrogate time-series were generated by cutting at a single point at a random location the original time series and exchanging the two resulting time courses (Aru *et al.*, 2015). We restricted the range of the selected cutting point in a temporal window of width equals to 10 sec in the middle of the recording session (between 25 – 35 sec). This surrogate scheme was applied to the original whole time series and not to the signal-segment at every slide. Repeating this procedure leads to a set of surrogates with a minimal distortion of the original phase dynamics, while the non-stationarity of the brain activity is less destroyed compared to shuffling the time series or cutting and rebuilding it in more than one time points.

The Delay vector variance (DVV) method uses predictability of the signal in phase space to characterize the time series. Using the surrogate data methodology, so called DVV plots and DVV scatter diagrams can be generated using the DVV method, as a test statistic, to examine the determinism/stochastisity and linearity/nonlinearity within a signal simultaneously. In DVV scatter diagram, the target variance values of the original signal is plotted against the averaged variance values, calculated over a number of iAAFT surrogates (Gautama et al., 2004). As a result, for linear signals, the scatter diagram coincides with the bisector line and conversely for nonlinear signals, the scatter diagram deviates from bisector line as shown in the S2.

In S2, we demonstrated how the method of creation of surrogates can affect their determinism and nonlinearity. S1.a demonstrates the degree of nonlinearity of the original time series deviated from the surrogates used in DVV method. S1.b shows the preservation nonlinearity in the surrogates created by cutting at a single point at a random location the original time series and exchanging the two resulting time courses (Aru et al., 2014). Contrary, the nonlinearity destroyed in surrogates created by shuffling the original time series. It is important to mention here that surrogates used in the DVV method are different compared to the surrogates used for creation a baseline for connectivity analysis.


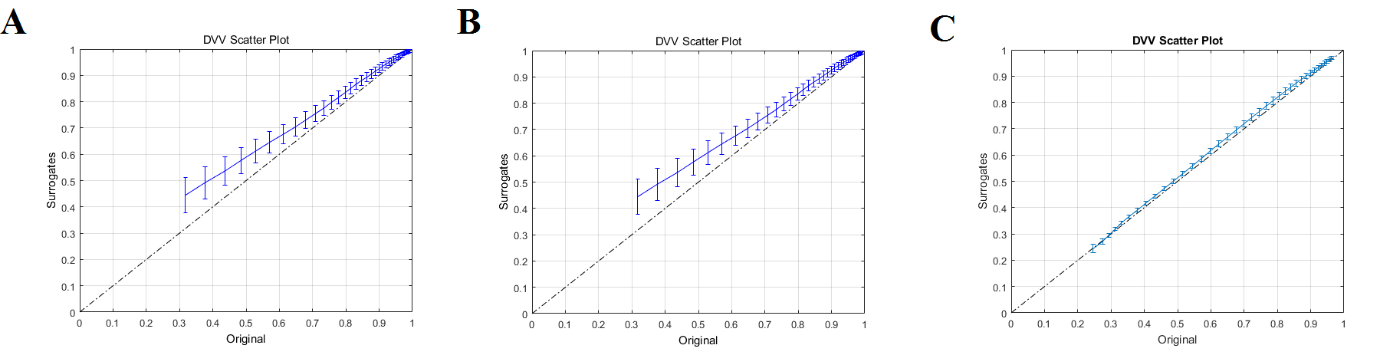


**S2. Estimated nonlinearity with DVV of original time series and the related surrogates using two approaches.**

1. Nonlinearity of original time series
2. Nonlinearity of surrogates using a random point
3. Nonlinearity of surrogates created by shuffling the original time series

Using the method of delay vector variance (DVV), we estimated the nonlinearity/nonstationarity of the surrogate time series which didn’t differ statistically with the original time series (see S1 in supp. material ;) (Gautama *et al.*, 2004). This procedure assures that the real and surrogate indices both have the same statistical properties. For each data set the surrogate iPLV (siPLV) was then computed. We then determined a one-sided *p*-value for each iPLV value that corresponded to the likelihood that the observed value could belong to the surrogate

distribution. This was done by directly estimating the proportion of ''surrogate'' iPLVs that were higher than the observed iPLV (Theiler *et al.*, 1992). The p-value reflected the statistical significance of the observed iPLV-level (a very low value revealed that it could not have appeared from processes with no iPLV coupling). The outcome of this procedure was to get 36 p-values per pair of sensors at every quasi-instantaneous

functional connectivity graph (FCG). Applying Bonferroni correction to correct for the 36 p-values for each pair of sensors and temporal segment, we can face three cases : a) only one frequency survived the statistical threshold e.g. the θ (S3.B.a) ), b) two survived the Bonferroni correction and in that case we selected the one with the maximum iPLV (S3.b) ) and c) none of the 36 frequencies survived in that case, we don’t assign any dominant type of interaction for that particular pair (S3B.c) ).


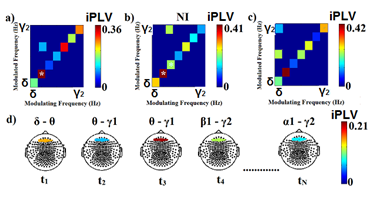


**S3. Defining the dominant coupling mode per pair of EEG sensors.**

At a second level, we applied FDR method (Benjamini, Hochberg, 1995) to control for multiple comparisons within each snapshot of the dynamic graph (FCG – a 64 x 64 matrix with tabulated p-values) with the expected fraction of false positives set to $q\leq0.01$. Finally, for each subject the resulting TVIiPLV profiles constituted to two 3D arrays of size [1856 (time windows) x 64 (sensors) x 64 (sensors)] with a value of 0 indicated a non-significant iPLV value . The first 3D graph keeps the weights from the iPLV estimator while the second 3D graph keeps an index from 1 up to 36 assigned to the dominant type of interaction (1 for δ, 2 for θ,….,8 for γ_2_, 9 for δ- θ,…,36 for γ_1_-γ_2_). The whole procedure is called statistical filtering approach.

**Section 4. A data-driven Topological Filtering Scheme based on Orthogonal Minimal Spanning Trees**

Our data-driven thresholding algorithm based on OMSTs works as followed:

a) We extract the OMSTs by applying iteratively the Kruskal's algorithm on the inversed functional brain graph because we want to collect the most significant connections under the constraint of MST.

b) After extracting the 1st MST, we substituted the V-1 edges with 'Inf' in the original network in order to avoid capturing the same edges and also to keep the orthogonality of the next MST.

c) We aggregated connections over the OMSTs (including the 1st) so as to optimize the formula global efficiency – cost versus cost. This procedure can employ e.g. 3*(V-1) edges from the first three OMSTs plus 10 edges from the 4th OMSTs.

d) For each adding connection, we estimated the objective function of Global Cost Efficiency (GCE) = global efficiency − cost) where cost denotes the ratio of the total weight of the existing edges divided by the total strength of the original full-weighted graph. The values of this formula range within the limits of an economical small-world network for healthy control participants (Bassett, Bullmore, 2006). Our criterion to topologically filter a given brain network is by finding the maximum value of the following quality formula:

$$J_{GCE}^{OMSTs}=GE -Cost (5)$$

A recent study based on original and artificial biological networks demonstrate that wiring cost supports the evolution of both modular and hierarchical organization of biological networks (Mengistu *et al.*, 2016). Additionally, these biological networks exhibit higher performance in terms of information flow and adoptability to new environments. Complementary to previous studies that demonstrate the relationship between sparsity and hierarchy (Corominas-Murtra *et al.*, 2013), this study explained and validated why sparsity leads to hierarchy under the force of wiring cost and provides new information about the evolution of hierarchy (Mengistu *et al.*, 2016). For that reason, both global efficiency as an index of how efficient the network operates and the cost should be part of the optimized function J.

OMST were presented to a static functional connectivity graph (FCG) within δ frequency band for eyes-open and eyes-closed. S4 demonstrates the first three OMST for both examples.


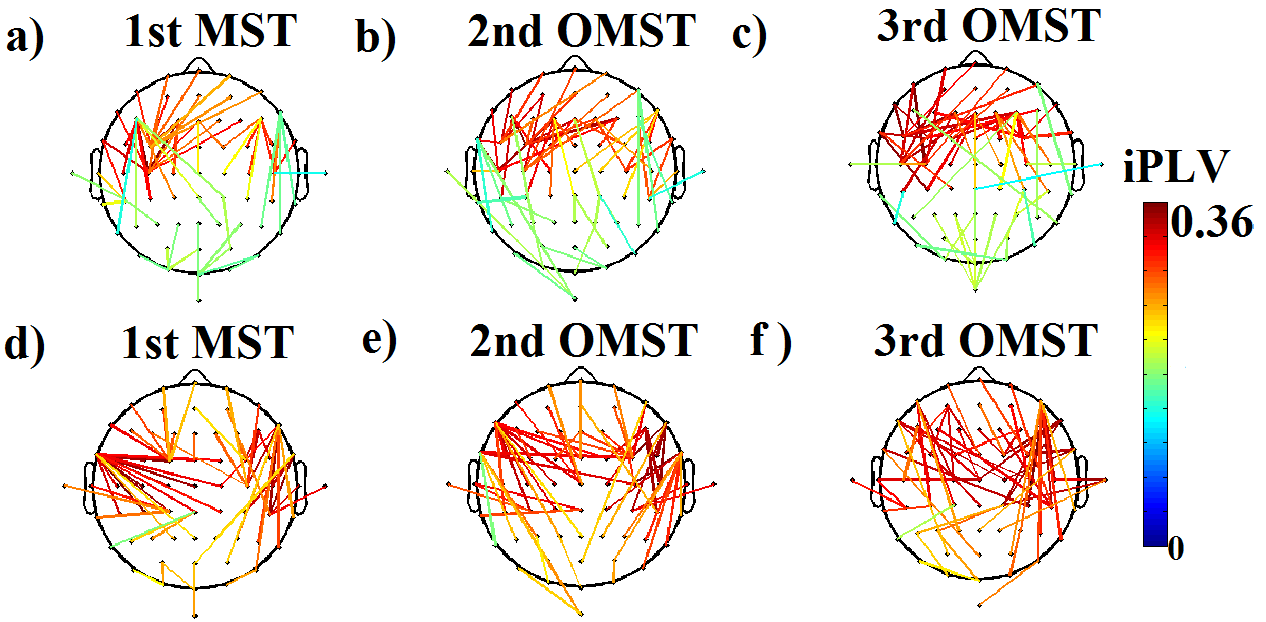


**S4**. An application of Orthogonal Minimal Spanning Tree (OMSTs) applied to a static functional connectivity graph (FCG) from δ frequency bands during eyes-open condition (a-c) and eyes-closed condition (d-f).

S5 demonstrates how our algorithm works in comparison with algorithm proposed in (Bassett, Bullmore*,* 2006) from a single subject at δ band from the eyes-open condition. The basic difference of these two algorithms is the sampling of connections from the given brain network. Our approach based on OMST while the one used by (Bassett, Bullmore*,* 2006), on iteratively absolute thresholding the weights of functional connections without any topological criterion and distinguishes weak from strong connections. The two curves represented the quality formula in (5) over various costs using the two sampling approaches.

Finally, we extracted as optimal thresholding functional brain network the one that maximizes the formula in (5) (see the arrow in S3). We can clearly see in S3 that OMST optimizes better the topological criterion compared to (Bassett, Bullmore, 2006).


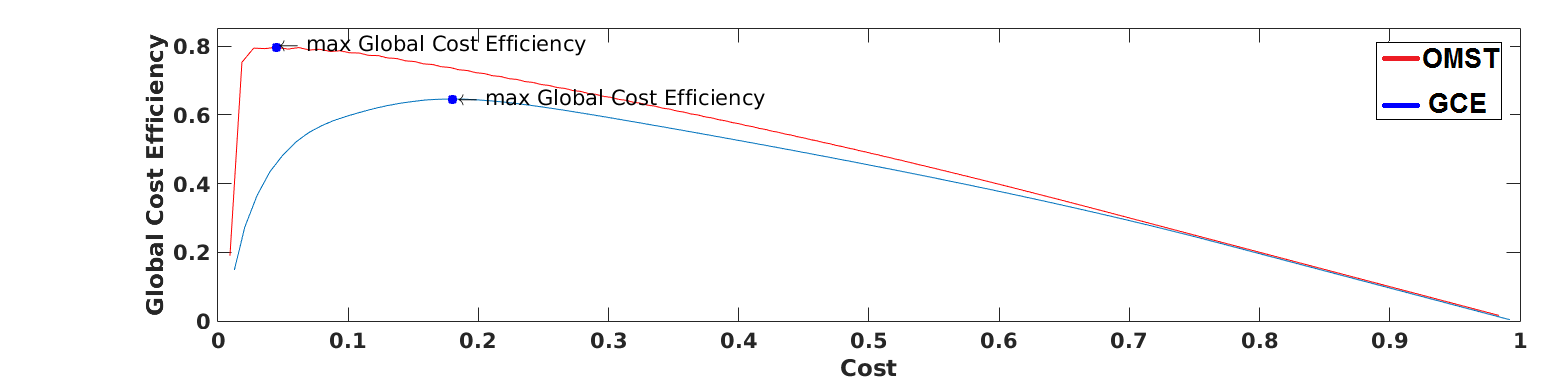


**S5.** A demonstration of how GCE algorithm (GCE) and the proposed Orthogonal Minimal Spanning Tree (OMST) data-driven thresholding scheme are working applied to a static functional connectivity graph (FCG) from δ frequency bands during eyes-open condition. Blue circles denote the maximization of global cost efficiency with the two approaches.

We applied the data-driven topological filtering scheme based on OMST at every quasi-instantaneous FCG from the dynamic IDFCG. After statistical and topological filtering approaches applied to the IDFCG, the derived IDFCG were fed to the proposed symbolization scheme.

**Section 5. Complexity of individual time series as symbolic sequences**

Next, a complexity index (CI) was estimated for each STS^NNMF-VQ^. CI quantifies the ‘richness of the language’ within a symbolic sequence, and has been used in several fields, such as data compression, data mining, computational biology computational linguistics (Leve et al., 2001). Low CI values describe sequences containing frequent repeated substrings that become periodic. The magnitude of the derived CI values was evaluated according to their deviation from the maximum complexity that can be derived by random versions of the original symbolic sequence. To achieve this CI values were z-score transformed using the standard deviation of the distribution of 1000 randomized versions of the original symbolic sequence.

In addition to determining the optimal number of distinct symbols describing the original time series, the length of potentially repeated patterns of symbols (i.e., substrings) in a symbolic sequence was parametrized. For example, for a symbolic sequence with k = 2 and length = 3, there are 2^3^=9 possible strings (111, 112, 121, 222,211, 212). Here, CI values were computed for substring sizes (word length) ranging between 3 and 20 symbols within each symbolic sequence (Janson et al., 2004).

**Section 6. The importance of a data-driven topological filtering scheme – the OMST approach**

To further enhance the importance of adopting and applying a proper topological filtering scheme, we compared the results presented in this study using the orthogonal minimal spanning trees (OMST) with three well-known arbitrary thresholding schemes. These are the absolute threshold, the mean degree and the density. As a proper absolute thresholding scheme, we estimated the median strength from all the subjects and across time and space (T=0.28). For mean degree, we selected k =5 while for density, we kept the 5 % of the strongest connections for each time-instant functional connectivity graph.

None of the aforementioned arbitrary thresholding schemes guarantee the connectiveness of the brain network. For that reason, we checked for disconnected node and in this case that we detected any, it is connected to the rest of network with its strongest connection.

Following the same strategy as in the main text, we attempted to demonstrate how the prediction of age and the classification of the two age groups is affected. Regarding the prediction of age, we couldn’t succeeded to fit a model linear and non-linear in the three arbitrary thresholding schemes. Complementary, the classification results were worse in both datasets and in both conditions for each of three cases (S.Table1.2).

**S.Tables**

|  | **Accuracy** | **Sensitivity** | **Specificity** |
| --- | --- | --- | --- |
| **Eyes-Open** | **54.34 %** | **53.17 %** | **54.67 %** |
| **Eyes-Closed** | **52.34%** | **54.41 %** | **55.01 %** |

**S.Table 1.a** Accuracy, sensitivity and specificity for the two-class classification problem  **for the training dataset based on absolute threshold.**

|  | **Accuracy** | **Sensitivity** | **Specificity** |
| --- | --- | --- | --- |
| **Eyes-Open** | **57.78 %** | **56.09 %** | **55.78 %** |
| **Eyes-Closed** | **55.66%** | **55.67 %** | **56.12 %** |

**S.Table 1.b** Accuracy, sensitivity and specificity for the two-class classification problem  **for the training dataset based on mean degree.**

|  | **Accuracy** | **Sensitivity** | **Specificity** |
| --- | --- | --- | --- |
| **Eyes-Open** | **59.19 %** | **58.28 %** | **57.64 %** |
| **Eyes-Closed** | **60.48%** | **58.79 %** | **59.12 %** |

**S.Table 1.c** Accuracy, sensitivity and specificity for the two-class classification problem  **for the training dataset based on density.**

|  | **Accuracy** | **Sensitivity** | **Specificity** |
| --- | --- | --- | --- |
| **Eyes-Open** | **56.57 %** | **55.61 %** | **55.48 %** |
| **Eyes-Closed** | **57.58%** | **56.37 %** | **57.81 %** |

**S.Table 2.a** Accuracy, sensitivity and specificity for the two-class classification problem  **for the testing dataset based on absolute threshold.**

|  | **Accuracy** | **Sensitivity** | **Specificity** |
| --- | --- | --- | --- |
| **Eyes-Open** | **59.61 %** | **58.63 %** | **58.24 %** |
| **Eyes-Closed** | **58.77%** | **56.82 %** | **57.91 %** |

**S.Table 2.b** Accuracy, sensitivity and specificity for the two-class classification problem  **for the testing dataset based on mean degree.**

|  | **Accuracy** | **Sensitivity** | **Specificity** |
| --- | --- | --- | --- |
| **Eyes-Open** | **60.83 %** | **59.61 %** | **59.31 %** |
| **Eyes-Closed** | **61.47%** | **60.21 %** | **59.81 %** |

**S.Table 2.c** Accuracy, sensitivity and specificity for the two-class classification problem  **for the testing dataset based on density.**

**References**

Aru J, J. Aru, V. Priesemann, M. Wibral, L. Lana, G. Pipa, *et al.*Untangling cross-frequency coupling in neuroscience**.** Curr Opin Neurobiol, 31C (2014), pp. 51–61

Bassett, DS., and Bullmore, E. (2006). Small-world brain networks. *Neuroscientist* 12, 512–523.

Benjamini, Y., and Hochberg, Y. (1995). Controlling the false discovery rate: A practical and Powerful Approach to Multiple Testing. *Journal of the Royal Statistical Society* 57, 289–

Buzsaki, G. (2010). Neural syntax: cell assemblies, synapsembles, and readers. *Neuron* 68, 362–85.

Buzsaki, G., Logothetis, N., Singer, W. (2013). Scaling brain size, keeping timing: evolutionary preservation of brain rhythms. *Neuron* 80, 751–764.

Canolty, R. T., and Knight, R. T. (2010). The functional role of cross-frequency coupling. *Trends in Cognitive Science* 14, 506-15, 2010.

Corominas-Murtra, B., Goni, J., Sole, R. V., and Rodriguez-Caso, C. (2013). On the origins of hierarchy in complex networks. *Proceedings of the National Academy of Sciences*, 13316–13321.

Dimitriadis SI, Laskaris NA, Tsirka V, Erimaki S, Vourkas M, Micheloyannis S, Fotopoulos S. A novel symbolization scheme for multichannel recordings with emphasis on phase information and its application to differentiate EEG activity from different mental tasks. *Cognitive Neurodynami*cs 2012a;6:107-113. **doi**:10.1007/s11571-011-9186-5

Dimitriadis SI, Laskaris NA, Tzelepi A. On the quantization of time-varying phase synchrony patterns into distinct functional connectivity microstates (FCμstates) in a multi-trial visual ERP paradigm. *Brain Topogr*, 2013a;26:397–409.

Dimitriadis, S. I., Laskaris, N. A., Bitzidou, M. P., Tarnanas, I., Tsolaki, M. (2015). A novel biomarker of amnestic MCI based on dynamic Cross-Frequency Coupling patterns during cognitive brain responses. (2015). *Frontiers in Aging Neuroscience* **9**:350. doi: 10.3389/fnins.2015.00350

Dimitriadis, S. I., Nikolaos, A. L., Panagiotis, G. S., Jack, M. F., and Papanicolaou, A. C. (2016). Greater Repertoire and Temporal Variability of Cross-Frequency Coupling (CFC) Modes in Resting-State Neuromagnetic Recordings among Children with Reading Difficulties. *Frontiers in Human Neuroscience* **10**:63. doi: 10.3389/fnhum.2016.00163

Dimitriadis,SI,Sun Y,Thakor,NV,Bezerianos A. Causal Interactions between Frontal^θ^ – Parieto-Occipital^α2^ Predict Performance on a Mental Arithmetic Task. [Front Hum Neurosci](https://www.ncbi.nlm.nih.gov/pmc/articles/PMC5022172/). 2016; 10: 454.Published online 2016 Sep 14. doi:  [10.3389/fnhum.2016.00454](https://dx.doi.org/10.3389%2Ffnhum.2016.00454)

Dimitriadis SI, Salis C, Tarnanas I, and Linden DE (2017). Topological Filtering of Dynamic Functional Brain Networks Unfolds Informative Chronnectomics: A novel data-driven thresholding scheme based on Orthogonal Minimal Spanning Trees (OMSTs). *Front. Neuroinform*. **11**:28. doi: 10.3389/fninf.2017.00028.

Gautama,T D.P. Mandic, and M. M. Van Hulle. **The delay vector variance method for detecting determinism and nonlinearity in time series.** Physica D, vol. 190, no. 3-4, pp. 167-176, 2004.

Janson,S, S. Lonardi, and W. Szpankowski, “On average sequence complexity,” Theoretical Computer Science, vol. 326, no. 1–3, pp. 213– 227, Oct. 2004

Lachaux, J. P., Rodriguez, E., Martinerie, J., and Varela, F. J. (1999). Measuring phase synchrony in brain signals. *Human Brain Mapping* 8, 194–208.

Leve F, Séébold P. Proof of a conjecture on word complexity. *Bull. Belg. Math. Soc 2001;* 8(2).

Mengistu, H., Huizinga, J., Mouret, J.-B., and Clune, Jeff. (2016). The Evolutionary Origins of Hierarchy. *PLoS Computational Biology* 12.

Nolte, G., Bai, O., Wheaton, L., Mari, Z., Vorbach, S., and Hallett, M. (2004). Identifying true brain interaction from EEG data using the imaginary part of coherency. *Clinical Neurophysiology* 115, 2292–2307.

Theiler, J., Eubank, S., Longtin, A., Galdrikian, B., Farmer, J. D. (1992). Testing for nonlineaity in time series the method of surrogate data. *Physica D* 85, 77-94.

Voytek, B., Canolty, R. T., Shestyuk, A., Crone, N. E., Parvizi, J., Knight, R. T. (2010). Shifts in Gamma Phase–Amplitude Coupling Frequency from Theta to Alpha Over Posterior Cortex During Visual Tasks. *Frontiers in Human Neuroscience* **4**:191. doi: 10.3389/fnhum.2010.00191
